# A cross-neutralizing antibody between HIV-1 and influenza virus

**DOI:** 10.1371/journal.ppat.1009407

**Published:** 2021-03-22

**Authors:** Chang-Chun D. Lee, Yasunori Watanabe, Nicholas C. Wu, Julianna Han, Sonu Kumar, Tossapol Pholcharee, Gemma E. Seabright, Joel D. Allen, Chih-Wei Lin, Ji-Rong Yang, Ming-Tsan Liu, Chung-Yi Wu, Andrew B. Ward, Max Crispin, Ian A. Wilson

**Affiliations:** 1 Department of Integrative Structural and Computational Biology, The Scripps Research Institute, La Jolla, California, United States of America; 2 School of Biological Sciences, University of Southampton, Southampton, England, United Kingdom; 3 Oxford Glycobiology Institute, Department of Biochemistry, University of Oxford, Oxford, England, United Kingdom; 4 Division of Structural Biology, University of Oxford, Wellcome Centre for Human Genetics, Oxford, England, United Kingdom; 5 Department of Chemistry, The Scripps Research Institute, La Jolla, California, United States of America; 6 Centers for Disease Control, Taipei City, Taiwan; 7 Genomics Research Center, Academia Sinica, Taipei City, Taiwan; 8 The Skaggs Institute for Chemical Biology, The Scripps Research Institute, La Jolla, California, United States of America; National Institutes of Health, UNITED STATES

## Abstract

Incessant antigenic evolution enables the persistence and spread of influenza virus in the human population. As the principal target of the immune response, the hemagglutinin (HA) surface antigen on influenza viruses continuously acquires and replaces *N*-linked glycosylation sites to shield immunogenic protein epitopes using host-derived glycans. Anti-glycan antibodies, such as 2G12, target the HIV-1 envelope protein (Env), which is even more extensively glycosylated and contains under-processed oligomannose-type clusters on its dense glycan shield. Here, we illustrate that 2G12 can also neutralize human seasonal influenza A H3N2 viruses that have evolved to present similar oligomannose-type clusters on their HAs from around 20 years after the 1968 pandemic. Using structural biology and mass spectrometric approaches, we find that two *N*-glycosylation sites close to the receptor binding site (RBS) on influenza hemagglutinin represent the oligomannose cluster recognized by 2G12. One of these glycan sites is highly conserved in all human H3N2 strains and the other emerged during virus evolution. These two *N*-glycosylation sites have also become crucial for fitness of recent H3N2 strains. These findings shed light on the evolution of the glycan shield on influenza virus and suggest 2G12-like antibodies can potentially act as broad neutralizers to target human enveloped viruses.

## Author summary

The isolation of broadly neutralizing antibodies from HIV-1 patients has provided valuable insights on the design of HIV-1 vaccines. Among them, one group consisting of anti-carbohydrate antibodies target *N*-glycans that compose the glycan shield on the Envelope surface glycoprotein (Env) of HIV-1. Similar to HIV-1, human H3N2 influenza viruses are highly glycosylated due to the accumulation of *N*-glycosylation sites over the past 50 years of natural evolution. Here, we demonstrate that an anti-HIV-1 antibody, namely 2G12, can neutralize human H3N2 viruses from the past 35 years. Comprehensive analyses by mass spectrometry and negative-stain electron microscopy reveal that oligomannose on two *N*-glycosylation sites near the receptor binding site are the targets of 2G12. Of note, the distance and disposition between the two glycans are the same as the equivalent oligomannose cluster on HIV-1 Env. Furthermore, mutational study shows that these two *N*-glycosylation sites are both required for survival of the recent H3N2 strains. Our data suggest that 2G12 can broadly neutralize non-HIV-1 human viruses by targeting oligomannose sugars, if of sufficient density and disposition on the viral surface. Since many viral surface antigens are highly glycosylated, it is worth evaluating 2G12-like antibodies as potential general therapeutics for human viral pathogens.

## Introduction

The discovery of broadly neutralizing antibodies (bnAbs) from infected HIV-1 patients has contributed to design and development of HIV-1 vaccine candidates and therapies [1–7]. Many of these antibodies have demonstrated remarkable potency and breadth against diversified HIV-1 strains and subtypes [8,9]. One unusual class is anti-carbohydrate antibodies that exclusively target the glycan shield on the HIV-1 envelope glycoprotein (Env) [8–10]. Although the glycan shield of HIV-1 Env acts as a protective barrier that prevents or hinders recognition of the immunogenic protein surface of HIV-1 Env by the humoral immune system, the overly dense glycan shield on HIV-1 compared to host proteins results in the glycans themselves now being targeted by antibodies [1,3,4,11,12]. Such anti-carbohydrate antibodies have been very rarely seen against other viral pathogens [13,14].

HIV-1 is not the only heavily glycosylated viral pathogen. Since causing the 1968 pandemic, human seasonal H3N2 influenza viruses have gradually become more highly glycosylated due to the accumulation of *N*-glycosylation sites on its major surface antigen, hemagglutinin (HA) [15–17]. These newly acquired *N*-glycosylation sites are mainly located on the receptor binding domain (RBD) of the HA globular head, which contains the major antigenic sites [15,16,18,19]. *N*-linked glycans can help mask or redefine epitopes and lead to antigenic drift [20]. The relentless antigenic drift of both glycan and protein components in the HA necessitates annual updates of the seasonal influenza vaccine. Previous studies have nevertheless indicated that accumulation of less processed oligomannose glycans on the HA could be a target for mannose binding lectin, thereby facilitating virus inhibition both *in vitro* and *in vivo* [21–24].

These glycan modifications therefore raised the question whether anti-carbohydrate antibodies could now neutralize human seasonal H3N2 influenza viruses. Anti-carbohydrate antibody, 2G12, was one of the first handful of neutralizing antibodies to be discovered against HIV-1 and was isolated from pooled sera of HIV-1 patients [1,2]. 2G12 neutralizes HIV-1 by binding to a cluster of high-mannose glycans (mannose patch) on gp120 of Env around a site that is now known as the N332/ V3 base epitope [12,25,26]. 2G12 is extremely unusual in that it forms a domain-swapped dimer between the heavy chain variable regions of its two Fab arms and can, therefore, bind to the Manα1-2Man disaccharide at the tips of up to four different high mannose glycans on the gp120 surface without any observable contacts to amino acids [11,27,28]. In this study, we demonstrate that 2G12 has the ability to neutralize human seasonal H3N2 viruses over the last three decades and investigate the mechanism of neutralization by structure biology, mass spectrometry, and evolutionary analyses of *N*-linked glycans on viral and recombinant HA proteins. A conserved and a newly acquired high mannose *N*-linked glycosylation site nearby the HA receptor binding site (RBS) both contribute to this broad neutralizing activity of 2G12 on influenza virus.

## Results

### *N*-glycosylation sites accumulate on human H3N2 HA during natural evolution

**Fig 1:**
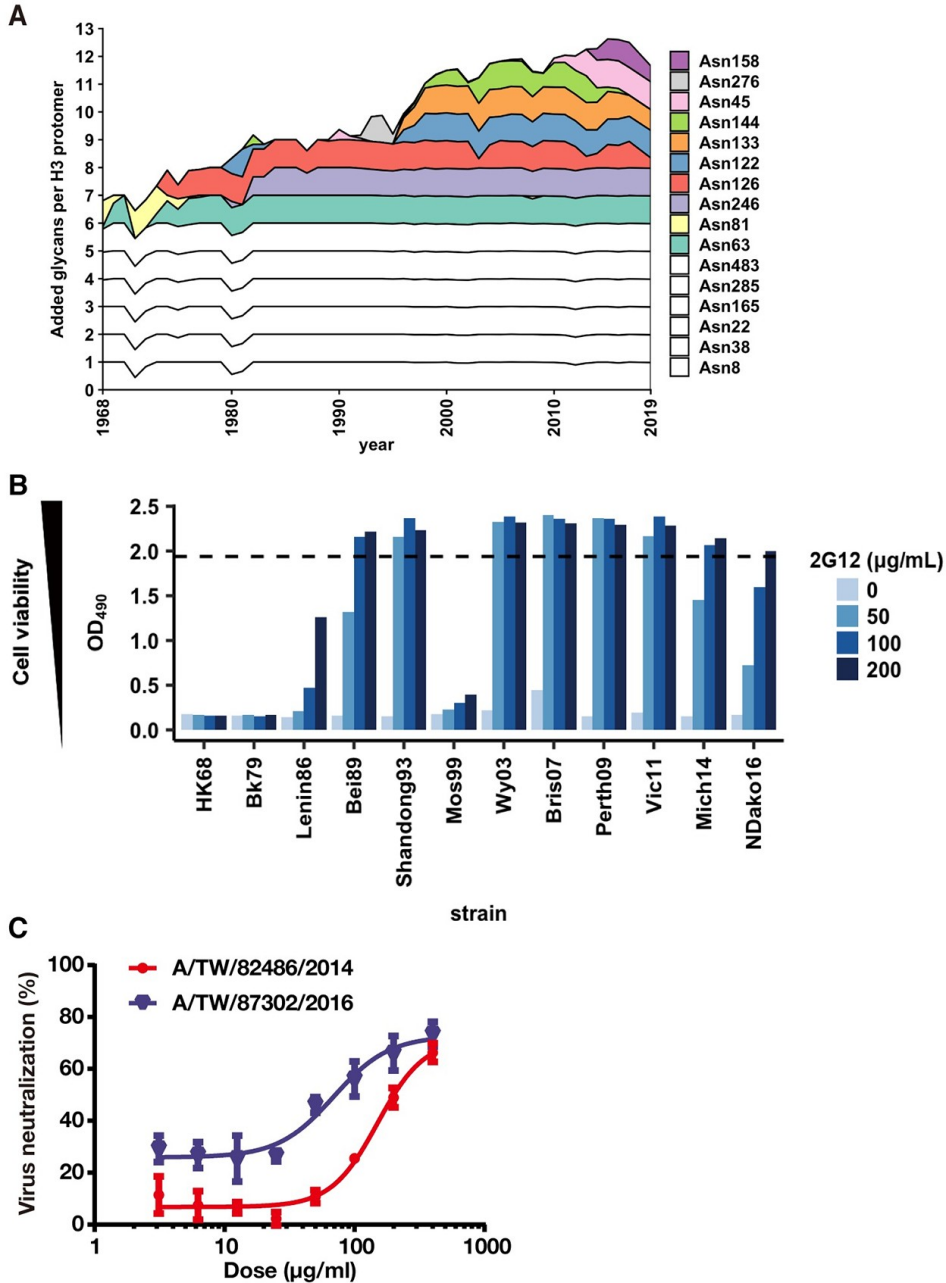
Evolution of *N*-glycosylation sites on H3 HA and broad neutralization of human H3N2 viruses from around 1990 by anti-glycan antibody 2G12. **(A)** The number of non-conserved *N*-glycosylation sites on HA has evolved from one in 1968 to seven in 2019 on HA1 as shown in the stack chart. Ten glycan sites are cumulatively involved, where some are retained and others substituted from 1968 to 2019. The six completely conserved *N*-glycosylation sites in human H3N2 viruses (N8, N22, N38, N165, N285 in HA1, and N154 in HA2, which corresponds to N483 in HA0) are shown in white. **(B)** Neutralization activity of 2G12 IgG with different H3N2 strains was measured by a cell viability assay at four different antibody concentrations. The OD_490_ value is proportion to the cell density. Dashed line represents the OD_490_ for cell only control with no virus. **(C)** Neutralization activity of 2G12 IgG against two clinical isolates. The IC_50_ values of 2G12 IgG to A/TW/82486/2014 and A/TW/87302/2016 are 151 and 69 μg/ml, respectively. The error bars on each value are the standard deviation of three technical triplicates.

The HA glycoprotein is formed by three homoprotomers, which are each composed of HA1 and HA2 from cleavage of HA0 by host cell proteases [29]. We analyzed the evolution of glycosylation sites on the HA since the emergence of the H3N2 1968 pandemic (Fig 1A). The number of *N-*glycosylation sites per HA protomer was six (five conserved and one non-conserved) when A/Hong Kong/1/1968 (HK68) first appeared in the human population in the 1968 H3N2 pandemic. Non-conserved *N-*glycosylation sites have since then accumulated from one to seven during five decades of H3N2 virus evolution (Fig 1A). Thus, the increased number and density of glycans on the HA during natural evolution from the 1968 pandemic suggests that high mannose glycans may be present on the HA.

### 2G12 broadly neutralizes human H3N2 viruses from the past thirty years

To test our hypothesis that human H3N2 viruses have acquired sufficient high mannose glycans to be a target for 2G12, we tested the neutralization activity of 2G12 on a panel of human H3N2 strains spanning from 1968 to 2016. 2G12 was able to neutralize most strains in the panel except for early isolates, such as HK68 and Bk79 (Fig 1B). The IC_50_’s were around 50–150 μg/ml and 2G12 was less potent compared to the broad stem neutralizer CR9114 by five to ten-fold, whereas the anti-malarial IgG311 showed no neutralizing activity against these viruses (S1 Fig) [30,31]. We also tested two strains from clinical isolates and found that they were also sensitive to neutralization by 2G12 (Fig 1C). To explore whether other anti-carbohydrate antibodies behaved similarly as 2G12, we further examined seven antibodies from HIV-1 infected individuals that involve binding of oligomannose (S1 Table) [12,32–37]. However, unlike 2G12, which recognizes only carbohydrate, these antibodies also interact with amino acids on HIV-1 Env. It was therefore perhaps not surprising that none of these antibodies exhibited neutralizing activity against H3N2 viruses (S2 Fig).

### Oligomannose-type glycans are enriched on the HA head in more recent H3N2 viruses

**Fig 2:**
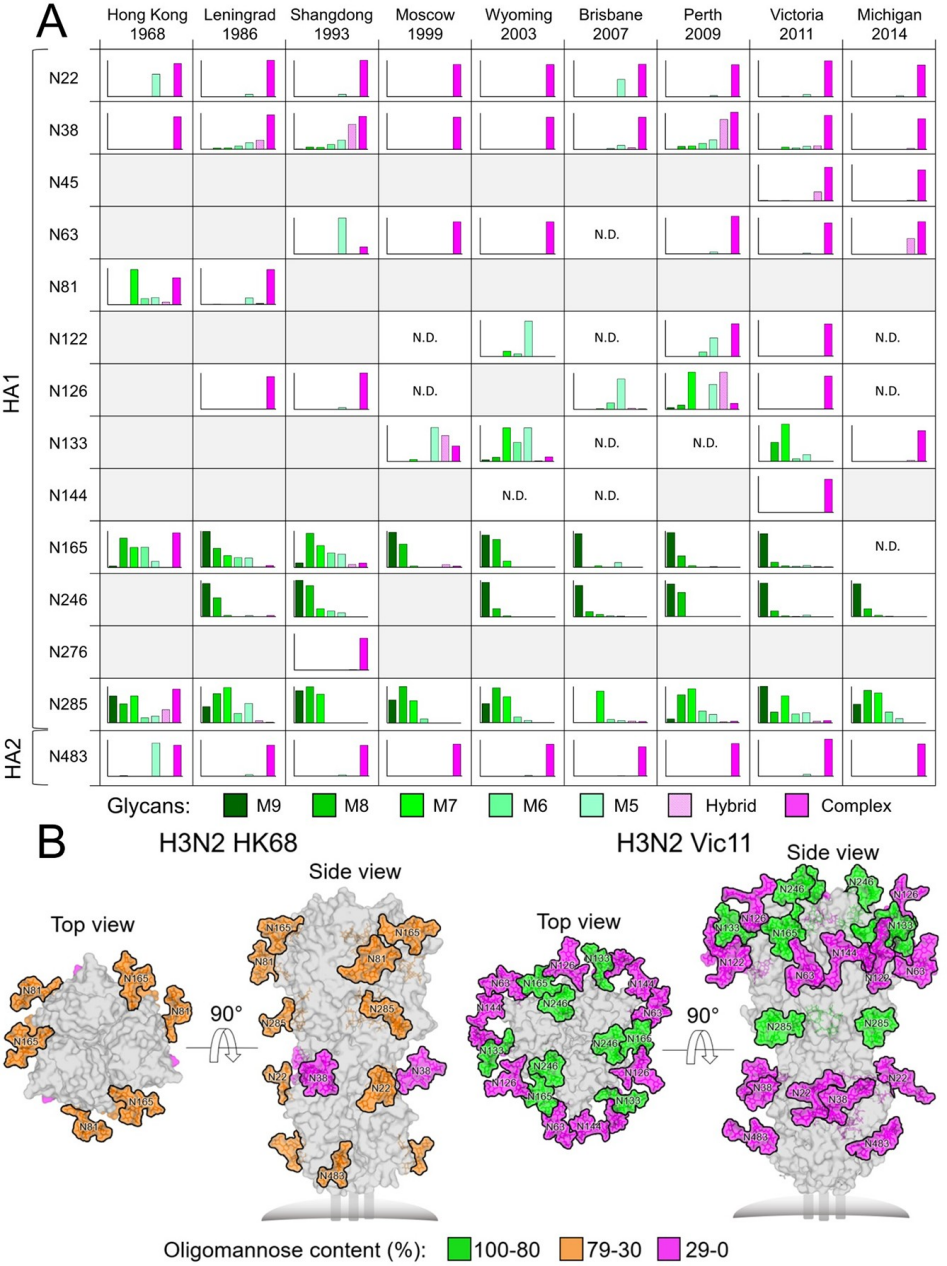
Site-specific *N*-linked glycosylation of H3N2 HA glycoproteins and models of fully glycosylated HK68 and Vic11 HAs. **(A)** Recombinant H3N2 HA glycoproteins were proteolytically digested and analyzed by LC-MS. Each graph summarizes the quantitative mass spectrometric analysis of the glycan populations at each individual *N*-linked glycosylation site, which are simplified into glycan categories. These categories are colored according to glycan compositions as per the key. The oligomannose-type glycoforms (M9 to M5; Man_9_GlcNAc_2_ to Man_5_GlcNAc_2_) are indicated in shades of green, hybrid-type glycans in dashed light pink, and complex-type glycans in pink. Glycosylation sites that are not present on individual HAs are shaded in grey, and sites where the glycosylation site is encoded, but glycan compositions could not be determined, are labelled as not determined (N.D.). **(B)** Experimentally observed glycans shown in Fig 2A are modelled onto the prefusion structure of trimeric HA from HK68 (PDB ID: 4FNK) [89] and Vic11 (PDB ID 4O5N) [15]. Glycans are colored according to the oligomannose-type glycan content as per the key, with the protein surface shown in grey.

We then profiled the glycoforms present on the HAs of different H3N2 viruses. Site-specific glycan compositions were delineated by liquid chromatography-mass spectrometry (LC-MS) of glycopeptides from recombinant HAs expressed in Expi293F cells (Fig 2A). HA samples were proteolytically digested using trypsin, chymotrypsin and GluC, and the resulting glycopeptides were analyzed by LC-MS. Five out of six glycosylation sites on HK68 displayed significant populations of both high mannose- and complex-type glycans, except for N38, which was fully complex. Interestingly, glycosylation sites on subsequent H3N2 strains diverged to either predominantly high mannose- or complex-type sites. These experimentally observed glycans were used to model fully glycosylated models of recombinant HAs of HK68 and Vic11 using crystal structures and glycan compositions revealed by the mass spectrometric analysis (Fig 2B). Increased occlusion of the HA protein surface by glycans has emerged since 1968, especially in the immunogenic globular head, with a cluster of high mannose glycans appearing at N165 and N246 in the HA head (Fig 2B).

We also sought to quantitatively assess the total compositions of the glycan structures displayed on H3N2 HAs. Enzymatically released glycans were fluorescently labelled and subjected to hydrophilic interaction chromatography-ultra-performance liquid chromatography (HILIC-UPLC) analysis (S3 Fig). Treatment with endoglycosidase H (Endo H) revealed the presence of under-processed oligomannose-type glycans across all the glycan sites. This analysis confirmed the abundance of trimmed high mannose glycans on HK68 HA, while more recent strains display the Man_8/9_GlcNAc_2_ 2G12 epitope. In order to ensure that the recombinant HAs recapitulate the glycans found on virally derived HAs, site-specific glycan analysis was also performed on HAs from HK68 and Vic11 viruses, grown in MDCK-SIAT1 cells, which represent 2G12-insensitive and 2G12-sensitive strains, respectively (S4 Fig). The site-specific glycan profiling from recombinant HAs corresponded well to the viral HAs. The high mannose glycans were particularly conserved between virus and recombinant material, with HAs on HK68 predominantly exhibiting the lower order high mannose glycans (Man_5-7_GlcNAc_2_) and HAs of Vic11 presenting predominantly larger Man_9_GlcNAc_2_ on N165 and N246.

### N165 is critical for 2G12 neutralization

**Fig 3:**
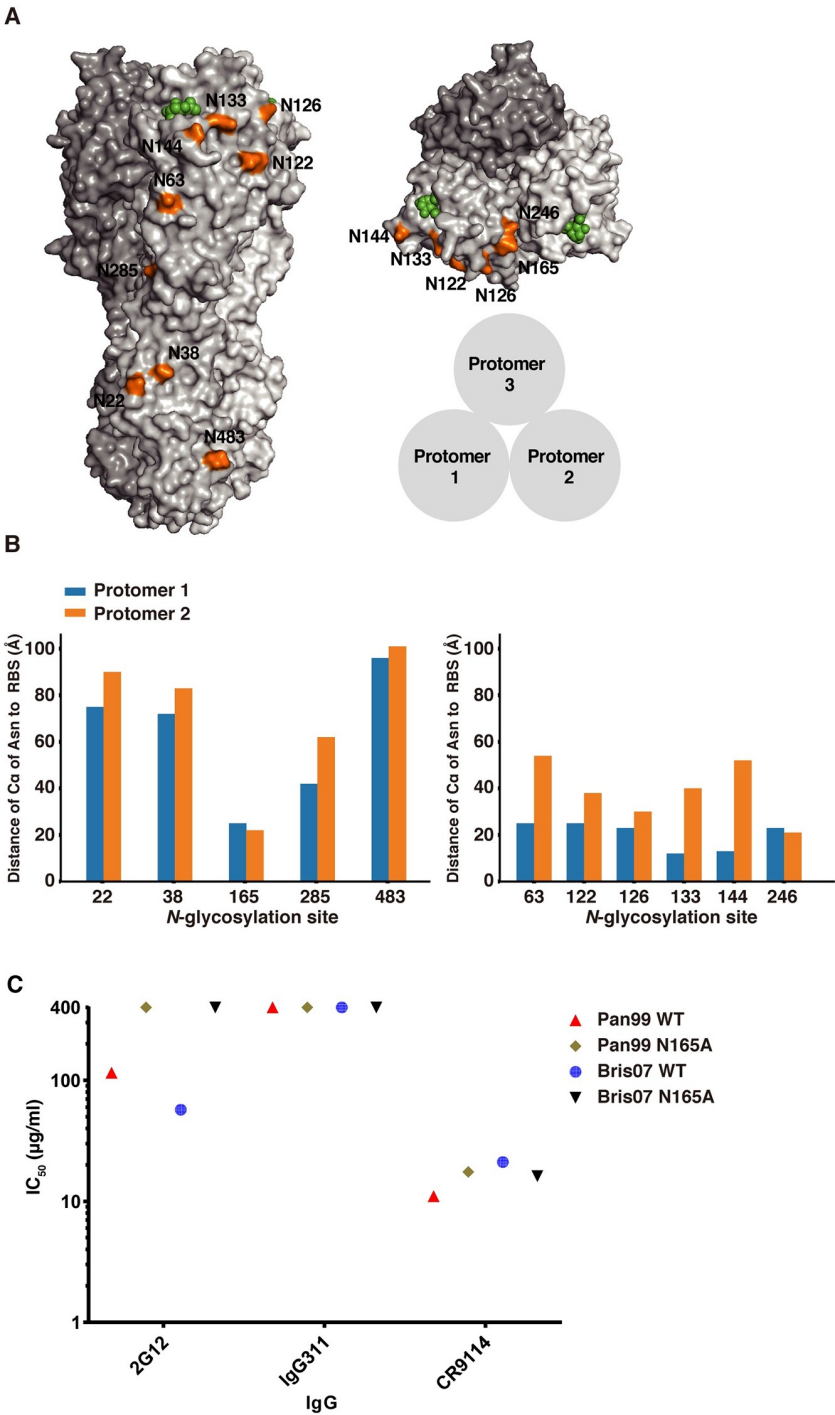
Evolution and spatial distribution of *N*-glycosylation sites on H3 HA and 2G12 neutralization. **(A)** Front and top views of *N*-glycosylation sites on A/Brisbane/10/07 (H3N2) HA (PDB 6AOV) [91]. Each *N*-glycosylation site is shown only on protomer 1 in orange. Human receptor analogue, 6’-sialylated di-N-acetyllactosamine (6´-SLNLN), in the receptor binding sites of protomers 1 and 2 of the trimer are in green. **(B)** The distance from C_α_ of the Asn in each *N*-glycosylation site to the sialic acid of the sialoside receptor in the receptor binding domain (RBD) on the same protomer (protomer 1), and the corresponding distance to the RBD of the neighboring promoter (protomer 2) among conserved *N*-glycosylation sites (left panel) and non-conserved *N*-glycosylation sites (right panel). **(C)** *In vitro* neutralization of wild type and N165A HA mutant Pan99 and Bris07 viruses by 2G12, CR9114, and IgG311. The IC_50_ of 400 μg/ml indicated that the antibody has no neutralizing activity against H3N2 viruses.

We then investigated the role of high mannose glycans in virus neutralization by 2G12. Many of the *N-*glycosylation sites are near the RBS of HA. For example, six non-conserved (N63, N122, N126, N133, N144, and N246) and one conserved (N165) *N*-glycosylation sites are less than 30 Å from the RBS within each protomer (Fig 3A and 3B). Since the glycan composition of the carbohydrate on N165 was determined to be high mannose here (Fig 2A) and in previous studies [38–40] and was located near the RBS, we investigated the role of N165 in 2G12 neutralization using human H3N2 influenza virus strains, A/Panama/2007/1999, which is close to A/Moscow/10/1999, and A/Brisbane/10/2007. While 2G12 neutralized A/Panama/2007/1999 and A/Brisbane/10/2007 with IC_50_ of 116 and 56 μg/ml, respectively, it could not neutralize their N165A mutants, which abolished the *N*-glycosylation site at residue 165 (Fig 3C). Thus, these data indicated that N165 is important for 2G12 neutralization. However, since N165 glycosylation site is conserved among all viruses in our H3N2 panel, it was not immediately clear why 2G12 did not neutralize early strains, such as HK68 and Bk79, which also contain the N165 glycosylation site, although the glycan composition does differ with respect to number of mannose moieties.

### N165 and N246 are both critical for 2G12 neutralization and virus fitness

**Fig 4:**
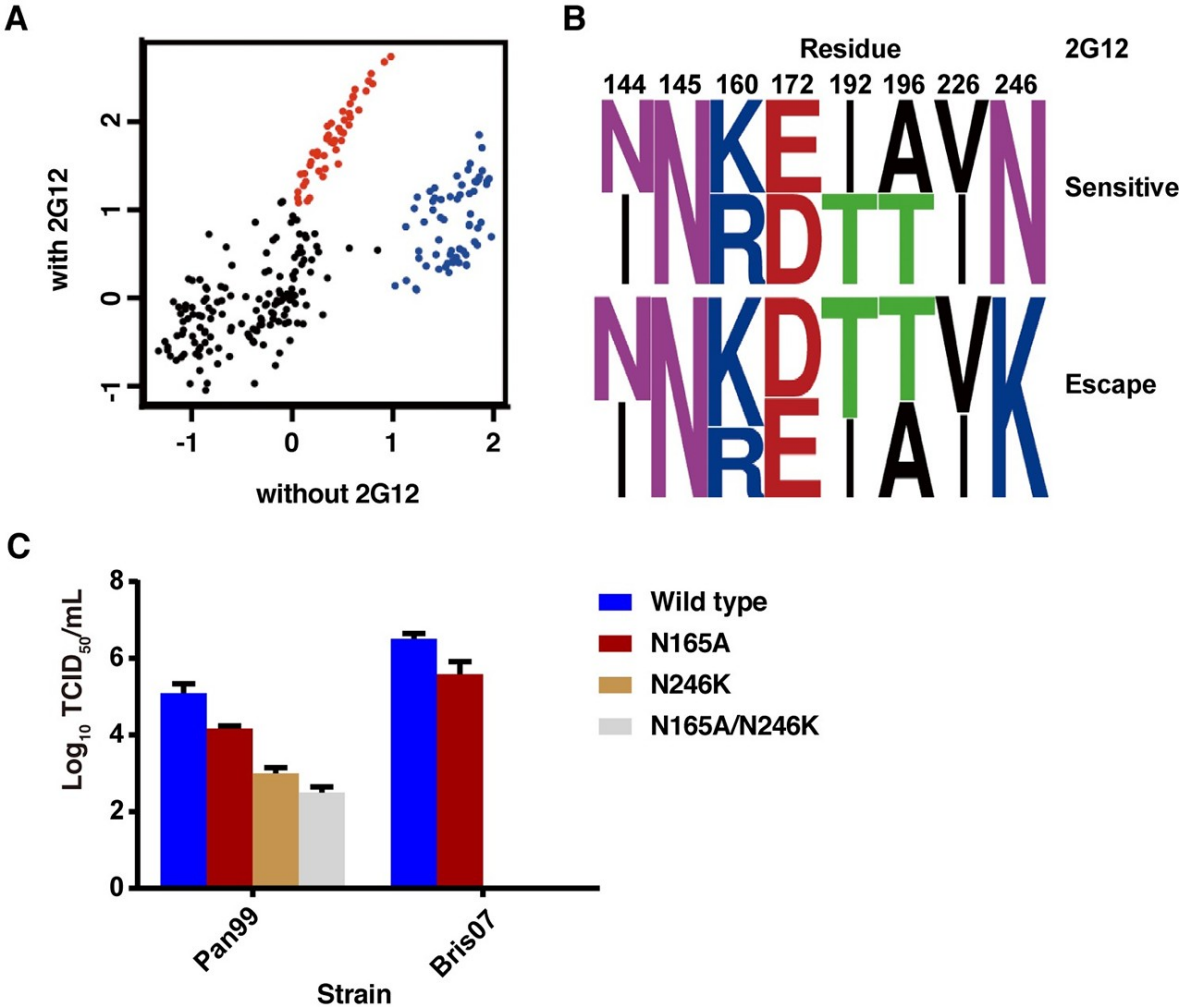
Virus escape mutants in a Pan99 library in the absence or presence of 2G12 and the fitness of N165/N246 mutations to Bris07 viruses. **(A)** The relative fitness of each of the 256 variants in the mutant library is shown. The x-axis and y-axis represent the relative fitness without and with 2G12 selection, respectively. Each data point represents one variant. The relative fitness of wild type is set as 0. Variants with a gain in relative fitness in the presence of 2G12 selection (escape mutants) are in red. Variants with a high fitness without 2G12 selection, which did not escape 2G12 are in blue. These two groups were selected for sequence logo analyses [81]. **(B)** Variants at specific sites (HA1 144, 145, 160, 172, 192, 196, 226, 246). The upper panel indicates residue combinations of wild-type variants with superior fitness, whereas the bottom panel indicates sequence preferences of 2G12 escape mutants. **(C)** The fitness effect of mutations N165A, N246K, and N165A/N246K on Pan99 and Bris07 HA were measured by median tissue culture infectious dose (TCID_50_). Error bars represent standard deviation of quadruple measurements.

It is noteworthy that, although 2G12 could neutralize H3N2 strains from 1986 onward in our panel, Mos99 was a striking exception (Fig 1B). To understand why Mos99 was able to escape from 2G12 neutralization, we also investigated a contemporaneous strain, Pan99, that could be neutralized by 2G12 (Fig 3C). We then focused on residues within the receptor binding domain (HA1 residues 117 to 265, H3 numbering) [41] that differ between Mos99 and Pan99. Eight differences are present within this region (S2 Table), including addition of two *N*-glycosylation sites at N144 and N246. We then designed a deep mutational scanning experiment to identify the key residues that differentiate 2G12-sensitivity between Pan99 and Mos99 (see Materials and Methods). Briefly, we constructed a Pan99 viral mutant library containing all possible combinations of HA mutations between Pan99 and Mos99 (S2 Table). The diversity of the viral mutant library was 2^8^ or 256 variants. The viral mutant library was passaged with or without 2G12 selection. The fitness of each variant in the mutant library was quantified and normalized to that of wild type, which was set as 0. In this analysis, we only focused on variants that have relative fitness of >1. A number of variants have a much higher relative fitness in the presence of 2G12 selection as compared to without 2G12 selection (red, Fig 4A). These variants likely represent 2G12 escape. We also identified a number of variants where increased relative fitness was comparable with and without 2G12 selection (blue, Fig 4A). These variants were 2G12-sensitive. Sequence logo analysis indicated that the only major difference between 2G12-escape variants and 2G12-sensitive variants was at residue 246, where 2G12-escape variants all have K246, whereas the 2G12-sensitive variants have N246 (Fig 4B). Overall, these data revealed that the new glycosylation site at N246 appeared to be required for 2G12 neutralization in addition to the N165 glycosylation site.

### The glycoform at N165 depends on the presence of N246

**Fig 5:**
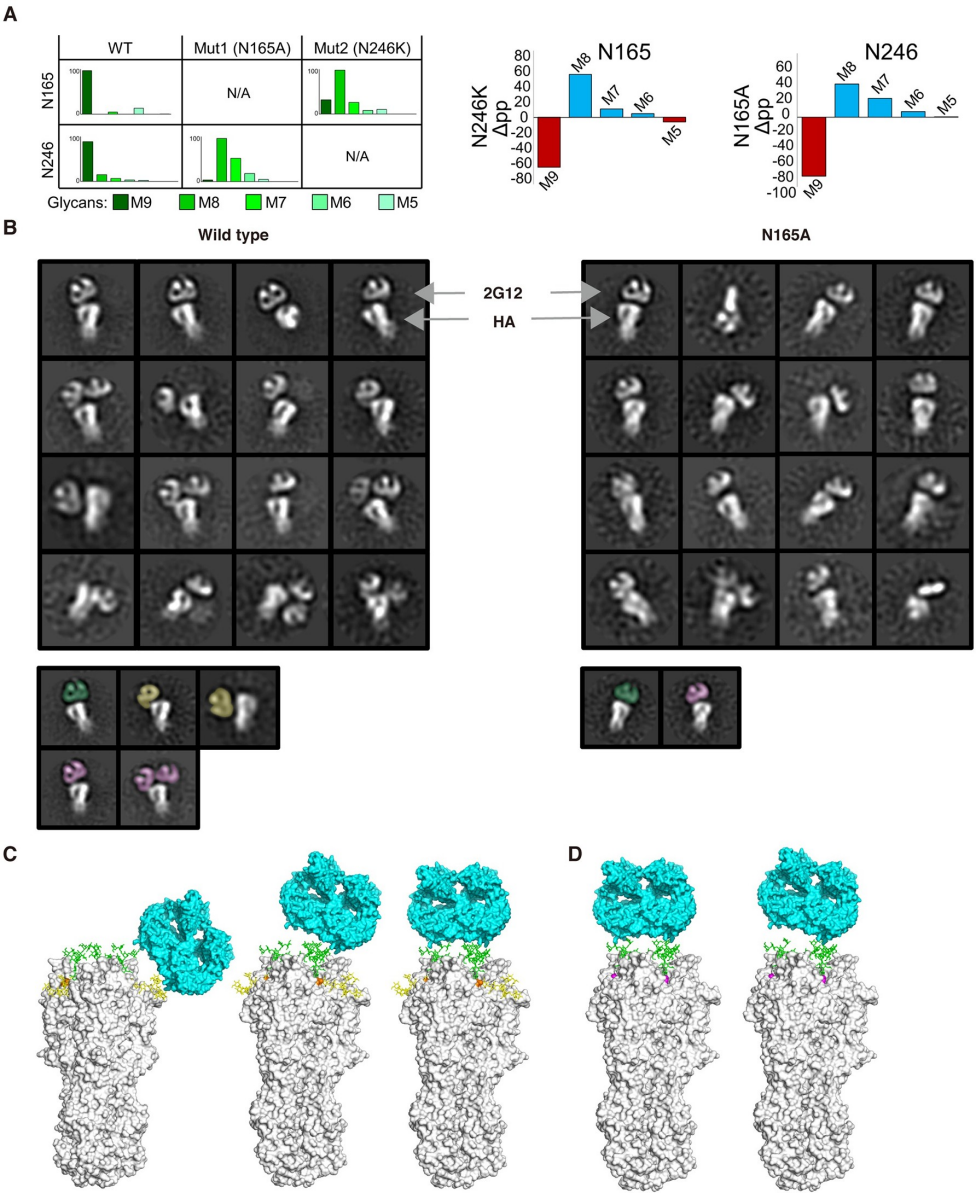
Glycoforms at N165 and N246 and 2G12 binding mode to HA wildtype and glycan mutants. **(A)** Site-specific glycosylation analysis of Bris07 HA glycan knockout mutants at N165 and N246 (left panel). Percentage differences in abundance of oligomannose-type glycans between glycan knockout mutants and WT Bris07 HA is also illustrated with decreased and increased abundance colored in red and blue (right panel), respectively. **(B)** Negative-stain electron microscopy (nsEM) images show the different modes of 2G12 recognition of Bris07 wild type (left panel) and N165A mutant (right panel) HA. Fab 2G12, as a domain-swapped dimer, and the HA trimer are indicated by arrows. 2G12 Fab involved in binding to two N246 glycans, one N246 glycan, and one N165 and possibly one N246 glycan are shown in green, purple, and yellow, respectively. Wild type and N165A HA are in white. **(C)** Front view of A/Brisbane/10/07 (H3N2) HA trimer (PDB 6AOV, in grey) [91]. Man_9_GlcNAc_2_ on N165 and N246 were modelled onto Bris07 wild type HA with Charmm-Gui [92]. 2G12 Fab dimer (PDB 6N2X, in cyan) [11] is shown bound to the lateral side (left panel) and apex of trimer in parallel (middle panel) or tilted (right panel) binding mode from the nsEM data in Fig 5B. Man_9_GlcNAc_2_ on N165 and N246 are in yellow and green, respectively. **(D)** Same as **(C)**, but N165A is shown in magenta. N246 was modelled with Man_8_GlcNAc_2_. 2G12 is shown bound to the apex of Bris07 N165A trimer as in the nsEM reconstruction. The Man_8_GlcNAc_2_ at N246 is in green.

We next investigated the contribution of these two *N*-glycosylation sites for 2G12 specificity and neutralization on another 2G12 sensitive virus. N165A, N246K, and N165A/N246K double mutants of Pan99 and Bris07 HA were constructed. For Pan99, the virus titer decreased by one to three-logs for N165A, N246K, and N165A/N246K double mutants compared to the wild type, respectively (Fig 4C). For Bris07, the titer of N165A was reduced about one log compared to the wild type and the N246K and the N165A/N246K double mutants could not be rescued (Fig 4C). These results were consistent with a previous study that showed that abolition of N165 attenuates virulence [42]. These data, therefore, showed that N246 is critical for Pan99 and Bris07 fitness, but is apparently not required in the early strains since the N246 glycosylation site is not present on or before 1979. Since N246 is essential for Bris07, we further examined the glycoforms at residues 165 and 246 of the wild type and N165A of Bris07 to delineate how they might functionally interact. LC-MS analysis revealed that wild type Bris07 presented predominantly Man_9_GlcNAc_2_ on both N165 and N246, whereas the N165A phenotype presented mainly Man_8_GlcNAc_2_ on N246. Likewise, the N246K mutant resulted in Man_8_GlcNAc_2_ being predominantly displayed on N165 (Fig 5A). Compared to the glycan composition of the wild type, the N165A mutation resulted in more changes in the glycoforms than the N246K mutation, such as at N22, N38, N126, and N285 (S5 Fig). N165 is located in the HA trimer interface [29], where its mutation may affect the stability and, hence, glycan processing of H3 HA trimers. This enhanced trimming of mannose residues, when glycan sites are knocked out, reveals structural constraints on the glycan-processing enzymes by the neighboring glycan site and consistent with similar mutational studies in HIV [27,43]. This glycan cluster is reminiscent of that found not only on HIV-1 Env, but also in the Lassa mammarenavirus glycoprotein complex (LASV GPC) and Middle East respiratory syndrome coronavirus (MERS-CoV) spike protein [27,44,45]. Furthermore, all of the 2G12-sensitive strains presented mainly Man_9_GlcNAc_2_ with some Man_8_GlcNAc_2_ on both N165 and N246 (Figs 2A and S3). These data suggested the presence of both N165 and N246 and particular high mannose glycoforms are crucial for neutralization by 2G12.

### Structural analysis of the 2G12 neutralization mechanism

We next investigated why 2G12 could neutralize wild type Bris07 but not its N165A mutant using negative-stain electron microscopy (nsEM). The nsEM analysis demonstrated that one to two 2G12 domain-swapped Fab dimers bound per wild-type HA trimer (Fig 5B). Notably, two major binding modes for this interaction were visualized on the globular head of the HA: one binding mode was to the apex on the HA trimer, whereas the other was to the lateral face of the trimer next to the RBS (Fig 5B and 5C). Furthermore, two major poses of 2G12 were found on the HA apex, with one being parallel to and the other tilted relative to the flat apex surface (Fig 5C). In contrast, 2G12 bound only to the apex of the HA N165A mutant in the parallel and slightly tilted binding modes (Fig 5B and 5D). As there was a visible gap between 2G12 and HA in the class averages (Fig 5B), 2G12 likely recognizes HA exclusively through antibody-carbohydrate interactions without involving the protein component of HA. According to the binding behavior of 2G12 to Bris07 N165A and WT in nsEM, we performed further biophysical analyses. Two apparent *K*_d_’s could be calculated in a 2:1 hetero-ligand binding model for both the 2G12-WT and 2G12-N165A interaction (S6 Fig). Both interactions can be represented by one lower affinity interaction (*K*_d_ = 286/212 nM), while the other was higher affinity at 40.9/43.5 nM for 2G12-WT and 2G12-N165A, respectively (S6 Fig). Combined with the nsEM images, the additional binding mode of the 2G12-WT, when both N165 and N246 are present, seems to account for most of the neutralizing activity for 2G12. Scrutinization of the spatial distribution of N165 and N246 on H3 HA trimer surface found that they are both located on adjacent strands of an antiparallel β-sheet with a distance between their C_α_’s of 4.6 Å (S7 Fig) within each HA protomer. Interestingly, the same secondary structural feature and C_α_ distances were found for two of the *N*-glycosylation sites on HIV-1 Env (N295 and N332, S7 Fig) that are involved in 2G12 recognition [1,11,46,47]. The distances between the ends of the flexible Man_9_GlcNAc_2_ on N165 and N246 within the same protomer was about 26 Å to 29 Å (S8 Fig). In addition, on the apex of HA trimer, the distance of Man_9_GlcNAc_2_ on N246 of one protomer to the next protomer was 14 Å (D1 to D1) and 44 Å (D3 to D3) (S8 Fig). Overall, the distance between each Man_9_GlcNAc_2_ on N165 to N246 in the same protomer or from Man_9_GlcNAc_2_ on N246 in different protomers matched the 2G12-Man_9_GlcNAc_2_ binding distances on gp120 [11]. Due to the flexible glycan interaction, the 2G12 domain-swapped Fab particle densities did not converge and 3D reconstructions could not be obtained. We modeled 2G12 binding to the HA using 2G12 Fab with PDB (6N2X) and HA structures from the PDB (6AOV) and fitted them into the different nsEM reconstructions derived from the class averages (Fig 5B). From this modeling study, we can conclude that the lateral binding pose involves N165 and N246 on the same protomer (Fig 5C). This interaction is lost in the N165A mutant, where binding is now only seen to the apex and mainly in the parallel pose (Fig 5B and 5D). Thus, we surmise that 2G12 in this mode binds to the high mannose glycans at N246 in different promoters of the trimer. The final pose is the more tilted apex orientation, where one or two glycans at N246 are involved, perhaps in a different combination to the parallel pose. The loss of 2G12 neutralization of N165A mutant HA suggests that the lateral interaction with both N165 and N246 is key for neutralization through blocking receptor binding compared to the apex-only interaction, but this is still not completely answered and may also depend on the exact glycoforms present at each site. In conclusion, during the natural evolution of human H3N2 influenza viruses from 1968 to present, the conserved N165 and emergent N246 formed a high-mannose cluster that conferred neutralizing activity of 2G12 on influenza virus.

## Discussion

Addition, removal or substitution of *N*-glycosylation sites of the glycan shield are one of the common strategies for RNA viruses to evade recognition by antibodies [20,48–50]. In this study, we demonstrated that 2G12, despite being elicited as an anti-HIV antibody, also has the ability to broadly neutralize human H3N2 influenza viruses that have evolved over the past five decades. Through comprehensive and evolutionary trajectory-based site-specific glycoproteomics, mutagenesis, and nsEM imaging, we found that neutralization by 2G12 is achieved through binding to two high mannose glycans present on *N*-glycosylation sites, N165 and N246, that are proximal to the HA RBS. 2G12 then likely represents a neutralizing antibody against human influenza viruses that only recognizes a glycan epitope on the HA. The emergence of N246 around 1980 endowed H3N2 viruses with an oligomannose cluster at N246 and N165 that can be recognized by 2G12. Escape mutants at these sites are then likely to have a high fitness cost, since abolition of the conserved N165 decreases viral fitness as shown here and in a previous study [42], and abolition of the N246 site could not be rescued in recent viruses.

How similar are the glycosylation profiles of antigens derived from virus with soluble recombinant proteins is one of the important issues for vaccine design [51–54]. For example, some differences in the glycoforms of soluble and viral HIV-1 Env have been found that may affect neutralization and elicitation of bnAbs [52, 53]. From our data on H3N2 influenza viruses, the site-specific high mannose glycans are conserved between recombinant HA from 293F cells and viral HAs derived from MDCK-SIAT1 cells (Madin-Darby canine kidney cells overexpressing the α-2,6-linked sialic acid receptor). This finding also implies that soluble recombinant HA could be used as an immunogen to attempt to elicit anti-carbohydrate antibodies. Although self-glycans are poorly immunogenic, if in high enough density on viral glycoproteins, they can become a focus of the immune response by antibodies that interact with both glycan and protein [4,35,55,56]. Multivalent binding to the high mannose glycans contributes to a potent IC_50_ of 2G12 against HIV-1. Notably, 2G12 exhibited neutralization (IC_50_ from 1 to 10 μg/ml) of sensitive HIV-1 viral strains through involvement of four major *N*-glycosylation sites that constitute the high mannose patch [26,57]. In our study, 2G12 neutralized sensitive H3N2 influenza strains only at sub-micromolar IC_50_ through targeting a mannose cluster that is formed by only two *N*-glycosylation sites, and is less potent compared to bnAb CR9114 by five to ten-fold. The 1 to 2-log difference seems to result from fewer high mannose *N*-glycosylation sites within the HA trimer compared to HIV-1 Env. Incomplete neutralization by 2G12 may be a consequence of other nearby N-glycosylation sites (N133, N285) that may contain ligands for 2G12, which distract from its neutralizing ability, e.g. 2G12 binding to wild type Bris07 HA has other minor poses that may include these glycans (Fig 5B). Heterogeneity in the glycan composition can also result in incomplete neutralization, especially for 2G12 if any complex glycans were present in addition to any heterogeneity in high mannose glycans. In a previous study, at low concentration (0.5 mM) of Man7, Man8, and Man9, 2G12 binding to gp120 was almost the same for Man9 and Man8 and less for Man7 (by ~20%) [58]. Incomplete neutralization is also found in several antibodies against HIV-1 possibly as a result of glycan heterogeneity [59–61].

**Fig 6:**
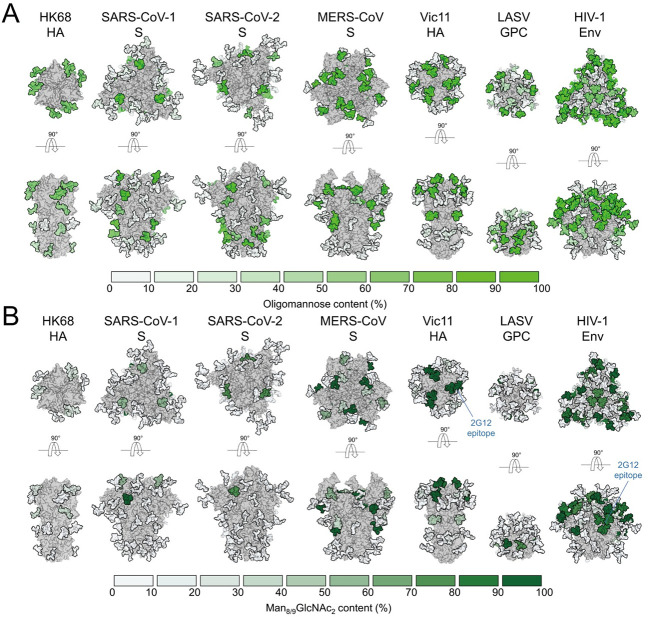
Differential under-processing of glycan shields in different enveloped viruses. **(A)** From left to right, HK68 HA, Severe acute respiratory syndrome coronavirus (SARS-CoV) spike, SARS-CoV-2 spike, MERS-CoV spike, Vic11 HA, LASV GPC, HIV-1 Env (PDB ID: 4FNK, 5X58, 6VSB, 5X59, 4O5N, 5VK2, 5ACO, respectively) [15,38,93–96]. Glycans are colored according to site-specific oligomannose-type glycan content [44,45], as per the key. **(B)** Glycans are colored according to Man_8/9_GlcNAc_2_ content (2G12 epitopes), as per the key.

Since 2G12-like antibodies are not typically found to be induced by influenza virus HA, it seems to indicate that the mannose cluster on the HA has not yet been a focus of immune selection pressure. Many enveloped virus pathogens, such as Ebola virus, Hepatitis C virus, Lassa fever virus and coronaviruses, including Severe acute respiratory syndrome coronavirus 2 (SARS CoV-2), are highly glycosylated and present oligomannose on their surface antigens (Fig 6). [44,54,62,63].

Indeed, besides H3N2 HAs and HIV-1 Env, 2G12 has been purported to bind SARS-CoV-2 S by recognizing high mannose glycans on the S2 subunit [64]. Mannose clusters on surface antigens can also be recognized or captured by various lectins, such as human mannose binding lectin (MBL) or DC-SIGN [65–67]. Similar to our finding of high mannose glycans near the RBS on H3 HA, some high mannose glycans are also found near the RBS of the glycoprotein of Lassa virus [44,54]. Based on our findings, it is possible that other clusters of high mannose glycans present near the critical functional sites of highly glycosylated viruses could be a target for 2G12. Furthermore, 2G12-like, Fab-dimerized glycan-reactive antibodies were recently isolated from an HIV-1 naïve population with a precursor frequency of 1 in 340,000. Such glycan-reactive antibodies were also found in rhesus macaques infected with simian-human immunodeficiency virus (SHIV), and Fab-dimerized glycan-reactive antibody precursors could be boosted through vaccination [68]. Thus, 2G12-like anti-carbohydrate antibodies without a domain swap can indeed be elicited through vaccination. Here, we reveal that 2G12 could neutralize human H3N2 viruses due to the emergence of an N246 glycosylation site around 30–35 years ago during H3N2 evolution from the 1968 pandemic. As 2G12 also binds to high mannose epitopes on HIV-1 Env, this raises the question whether such carbohydrates could act as a universal epitope on enveloped RNA viruses. The ontogeny of 2G12 has not been documented and most antibodies against viruses that have carbohydrate in their epitopes to date have been found in patients with chronic HIV-1 infection. While 2G12-like Fab-dimerized glycan antibodies and precursors have been discovered in HIV-1-naïve human subjects [68], further vaccination studies would shed light on eliciting 2G12-like anti-carbohydrate antibodies against highly glycosylated viruses.

## Materials and methods

### Sequence and structural analyses

A total of 55,135 human H3N2 HA protein sequences were obtained from Global Initiative on Sharing All Influenza Data (GISAID) from 1968 to 2019 (https://www.gisaid.org/). Sequences were aligned with MAFFT and cleaned [69]. Potential *N*-glycosylation sites (NXT/S, where X cannot be proline) were extracted with Biopython module and a stack graph of *N*-glycosylation sites on each HA among years was output in R [70]. To calculate the distance between each *N*-glycosylation site and the RBS, PDB: 6AOV was used and the distance from the Cα of each asparagine to the centroid of the sialic acid of the bound 6’-sialylated di-N-acetyllactosamine (6´-SLNLN) in the RBS was measured in PyMOL [71].

### Virus generation, passage and site-directed mutagenesis

To investigate the potency and breadth of anti-carbohydrate IgGs against human H3N2 influenza viruses, a panel of 13 H3N2 strains (from 1968 to 2016, S3 Table) were generated, recovered and titrated as in previous studies [39,72]. Briefly, these viruses were assembled on a WSN background with the HA gene from each tested H3N2 strain, the HK68 NA gene, and the remaining six gene segments from WSN. The eight genes were constructed in pHW2000 plasmids and transfected and recovered in a co-culture of 6:1 of human 293T cells and MDCK-SIAT1 cells. The Pan99 and Bris07 HA (N165/N165A) were used to generate virions for evaluation of the IC_50_ of 2G12, CR9114, and IgG311. To validate the neutralizing activity of 2G12 IgG on clinical strains, two strains A/Taiwan/82486/2014 and A/Taiwan/87302/2016, from influenza surveillance were kindly provided by Taiwan Centers for Disease Control [73,74]. To further understand which *N*-glycosylation site on HA conferred the neutralizing activity for 2G12 IgG, three mutants (N165A, N246K, and N165A/N246K) of A/Panama2007/1999 and A/Brisbane/10/2007 were generated using QuikChange XL Mutagenesis kit (Stratagene). All reverse genetics-derived and clinical strains were amplified in one passage in MDCK-SIAT1 cells and the TCID_50_ was measured with the Reed and Muench method [75]. The TCID_50_s of Pan99 and Bris07 wild type, N165A, N246K, and N165A/N246K were plotted with Prism 7.0 (Graphpad software).

### IgG expression and purification

The heavy and light chains of each anti-carbohydrate antibody, one anti-influenza virus antibody (CR9114), and one anti-malarial antibody (IgG311) (S1 Table) were cloned into phCMV3 vector which contained an N-terminal secreting signal peptide, and its C-terminus was fused to human IgG1 C_H_1 to C_H_3 domains for the heavy chain. All IgG1 were expressed in ExpiCHO expression system (Thermo Fisher Scientific) for two weeks to reach maximum yield according to the manufacturer user’s guide. The supernatant was harvested and filtrated according to manufacturer’s manual and further purified by the 5 ml HiTrap Protein G HP antibody purification columns. The elutes were neutralized with 0.1 M glycine buffer (pH 2.8), buffer exchanged into 1× Dulbecco’s phosphate-buffered saline (DPBS) and concentrated with Amicon Ultra-15 centrifugal filter (30-kDa cut-off) for microneutralization assay.

### Microneutralization

Measurement of neutralizing ability of 2G12 and other seven anti-carbohydrate binding antibodies was performed with microneutralization as described [76]. Briefly, around 5,000 MDCK-SIAT1 cells were plated in 96-well plates with 100 μl Dulbecco’s Modified Eagle Medium (DMEM) supplemented with 1× non-essential amino acids (NEAA), 1× penicillin-streptomycin and 10% fetal bovine serum (FBS) and incubated overnight at 37°C, 5% CO_2_ incubator. Cells were washed twice with DPBS. 100 TCID_50_ H3N2 viruses were co-incubated with 200 μg/ml anti-carbohydrate IgG in 100 μl Opti-MEM diluent containing 0.8 μg/ml N-tosyl-L-phenylalanine chloromethyl ketone (TPCK)-treated trypsin. After a one-hour incubation at 37°C, the mixture was transferred to the cell plate and incubated at 37°C, 5% CO_2_ for 72 hr. Cell viability was measured using the CellTiter 96 AQueous One Solution cell proliferation assay (Promega) according to the manufacturer’s manual as in [76]. For 2G12, CR9114, and IgG311 were further 2-fold serially diluted from 400 μg/ml to obtain the IC_50_ (50% maximal inhibitory concentration). For Bris07 WT, N165A and clinical strains, the IC_50_ was determined by enzyme-linked immunosorbent assay (ELISA) in triplicate [77]. The IC_50_ values were calculated by four-parameter nonlinear fit model and plotted with Prism 7.0 (Graphpad software).

### Recombinant HA expression and purification

Preparation of recombinant HA for nsEM and mass spectrometry was as previously described [78,79]. The HA ectodomain in H3 numbering (11 to 329 in HA1 and 1–176 in HA2, respectively) was fused with an N-terminal secreting signal peptide and followed by a C-terminal BirA biotinylation site, thrombin cleavage site, T4 trimerization domain, and Hisx6 tag in a phCMV3 vector. Different HA plasmids were transfected into Expi293F cells to express soluble recombinant HAs. Incubation conditions and harvest procedures were performed according to manufacturer’s guide (Thermo Fisher Scientific). HA proteins generated from Expi293F cells were purified by Ni Sepharose excel histidine-tagged protein purification resin (GE Healthcare), buffer exchanged with 20 mM Tris-HCl pH 8.0 and 150 mM NaCl. All HAs were treated with trypsin and purified by size exclusion chromatography (SEC) using a Hiload 16/90 Superdex 200 column (GE Healthcare) in 20 mM Tris pH 8.0, 150 mM NaCl, and 0.02% NaN_3_.

### Construction of A/Panama/2007/1999 HA mutant library

The method of library preparation was as described previously [80]. Briefly, the A/Panama/2007/1999 HA mutant library was generated by multiple overlapping PCR reactions (S4 Table). The short inserts with differences among the receptor binding domain (residue 117 to 265, in H3 numbering) of both Mos99 and Pan99 (256 combinations) on HA were amplified and assembled at equal molar into final library insert. All library inserts and vectors were subjected to PCR with BsmBI cutting sites at both the 5´ and 3´ ends. Primers are summarized in S4 Table. Vectors and inserts were then digested by BsmBI (New England Biolabs) and ligated using T4 DNA ligase (New England Biolabs). The ligated product was purified with NucleoSpin Gel and PCR Clean-up kit (Clontech Laboratories) and transformed into MAX Efficiency DH10B cells (Thermo Fisher Scientific), plated and incubated at 30°C overnight. About two million colonies were collected. The library plasmids were purified from the colonies with NucleoBond Xtra Maxi Kit according to manufacturer’s manual (Clontech Laboratories).

### Deep mutational scanning experiments

Virus mutant libraries were rescued in HEK 293T/MDCK-SIAT1 cells co-culture in a 6:1 ratio in T75 flasks (Corning). Virus libraries were harvested at 3-day post transfection and titrated. To test the fitness of viral mutant libraries at with or without 2G12 IgG, the viral libraries with a multiplicity of infection (MOI) = 0.0005 were incubated with or without 400 μg/ml 2G12 IgG in 37°C incubator for one hour. MDCK-SIAT1 cells in T75 flasks were washed twice with PBS and infected with MOI of 0.0005 in Opti-MEM medium supplemented with 0.8 μg/ml TPCK-treated trypsin for two hours. Infected cells were then washed twice with DPBS. Fresh Opti-MEM medium supplemented with 0.8 μg/ml TPCK-treated trypsin with or without 400 μg/ml 2G12 IgG was added to the cells. Supernatants were harvested at 24 hours post-infection. Two replicates were performed for both transfection and virus infection independently. Viral RNA was then extracted from the viral culture with QIAamp Viral RNA Mini Kit (Qiagen). The extracted RNA was then reverse transcribed into cDNA by Superscript III reverse transcriptase (Thermo Fisher Scientific). The cDNA was then amplified by PCR to add part of the adapter sequence and barcoding sequences required for Illumina next-generation sequencing (NGS) (A/Panama/2007/1999 HA mutant library). Illumina next-generation sequencing was performed with the MiSeq PE300 system at The Scripps Research Institute.

### Sequencing data analysis

Sequences handling from next-generation sequencing libraries were described as previously [80]. A paired-end read was filtered and removed if the corresponding forward and reverse reads did not match the residues designed or were low-quality reads. Mutants were counted by comparing every paired-end read to the wild type reference. Relative fitness was calculated for each mutant [76] and by the following formula:

$$Relative\;fitness={Log}_{10}\frac{\left({Numbers}_{mutant,post‐selection}+1)/({Numbers}_{mutant,input}+1\right)}{\left({Numbers}_{WT,post‐selection}+1)/({Numbers}_{WT,input}+1\right)}$$

For a given mutant, Numbers_mutant,post-selection_ represents the number of paired-end reads in post-selection mutant library, whereas Numbers_mutant,post-selection_ represents the number of paired-end reads in the input plasmid library. Numbers_WT,post-selection_ and Numbers_WT,input_ represent the number of paired-end reads that corresponds to wild types in the post-selection mutant library and input plasmid mutant library, respectively. A pseudonumber is added to all count items to prevent the denominator being zero. Both high fitness of 2G12 sensitive and resistant variants were selected for sequence logo analyses [81]. Custom python scripts for deep mutational scanning data analysis have been deposited to https://github.com/wchnicholas/Pan99_2G12_escape.

### Fab generation and negative-stain electron microscopy

2G12 Fab was generated from IgG with IdeS (Genovis) digestion in buffer containing 100 mM Bis-Tris pH 6.5, 150 mM NaCl. The reaction was incubated at 37°C for 2 hours followed by SEC by Hiload 16/90 Superdex 200 column (GE Healthcare) in 20 mM Tris pH 8.0, 150 mM NaCl. To form immune complexes, HA and 2G12, as a domain-swapped Fab dimer, were incubated at a 1:3 ratio of HA to 2G12 for 2 hours at room temperature. To prepare negative-stain EM grids, immune complexes at ~30 μg/ml were deposited onto glow-discharged carbon-coated 400 mesh copper grids (Electron Microscopy Sciences, EMS) and stained with 2% w/v uranyl formate. Grids were imaged on a 200kV Tecnai T20 transmission electron microscope (FEI) with an Eagle CCD 4k camera (FEI) at 62,000x nominal magnification. Micrographs were collected using Leginon [82], particles were picked and extracted using Appion [83], and particles were categorized into reference-free 2D class averages using Relion [84,85]. We were unable, however, to get convergence to achieve higher resolution 3D reconstructions. The putative binding interactions between 2G12 Fab and N165 and N246 of wild type Bris07 HA, N246 of Bris07 N165A HA were fitted based on the negative-stain EM and the binding features of 2G12 to oligomannose identified in a previous HIV-1 structural study [11].

### Biolayer interferometry binding assay

The binding affinity of 2G12 IgG to H3 HAs was measured by biolayer interferometry (BLI) with Octet RED96 system (ForteBio) according to a previous study [86]. Briefly, His-tagged trimeric recombinant HA of Bris07 WT and N165A (50 μg/ml) prepared in 1× kinetics buffer (0.01% BSA and 0.002% Tween 20 in 1× PBS) was loaded onto Ni-NTA biosensors. Sensors without loading were used as the baseline for background subtraction. Briefly, the assay consisted of five steps: (1) baseline: 60 secs with 1× kinetics buffer; (2) loading HA0 for 3 mins; (3) repeat step (1); (4) association: 2 mins with 2G12 IgG; and (5) dissociation: 2 mins with 1× kinetics buffer. K_d_, Data were fitted with 2:1 hetero-ligand binding mode for both Bris07 WT and Bris07 N165A to determine K_d_.

### Purification of H3N2 influenza viruses

Viruses were purified as described previously [87]. Briefly, A/Hong Kong/1/1968 and A/Victoria/361/2011 viruses were grown in MDCK-SIAT1 cells in 25 ml of Opti-MEM with 0.8 μg/ml TPCK-treated trypsin in T175 flasks in 37°C, 5% CO_2_ incubator. Supernatant was harvested after 48–72 hrs and centrifuged at 2000 rpm for 15 mins at 4°C and filtered with a 0.45 μm vacuum filter. Following filtration, around 200 ml medium was transferred to six 38.5 ml, thin-wall ultracentrifugation tubes (Beckman Coulter). The viral particles were pelleted down with SW28 swinging-bucket rotor at 18,000 rpm for 3 hrs at 4°C. The supernatants were aspirated, and the pellets were then resuspended in 1 ml of DPBS. 30 μl of the concentrated viruses without reduction was used for SDS-PAGE analyses. The gels were stained with instantBlue (Expedeon), and the band corresponding to molecular weight for HA0 was sliced and shipped for gel extraction, protease digestion, and glycoproteomic analyses.

### Release and labelling of *N*-linked glycans

Excised HA gel bands were washed alternately with acetonitrile and water before drying in a vacuum centrifuge. The bands were rehydrated with 100 μl of water and incubated with PNGase F at 37°C overnight. Aliquots of released *N*-linked glycans were also fluorescently labelled with procainamide, by adding 100 μl of labelling mixture (110 mg/ml procainamide and 60 mg/ml sodium cyanoborohydrate in 70% DMSO and 30% glacial acetic acid) and incubating for 4h at 65°C. Procainamide labelled glycans were purified using Spe-ed Amide 2 columns (Applied Separations).

### Glycan analysis by HILIC-UPLC

Labelled glycans were analyzed using a 2.1 mm x 150 mm Acquity BEH Glycan column (Waters) on an Acquity H-Class UPLC instrument (Waters), with fluorescence measurements occurring at λ_ex_ = 310 nm and λ_em_ = 370 nm. The following gradient was used: time (t) = 0: 22% A, 78% B (flow rate = 0.5 ml/min); t = 38.5: 44.1% A, 55.9% B (0.5 ml/min); t = 39.5: 100% A, 0% B (0.25 ml/min); t = 44.5: 100% A, 0% B (0.25 ml/min); t = 46.5: 22% A, 78% B (0.5 ml/min), where solvent A was 50 mM ammonium formate (pH 4.4) and B was acetonitrile. Quantification of oligomannose-type glycans was achieved by digestion of fluorescently labelled glycans with Endo H, and clean-up using a PVDF protein-binding membrane (Millipore). Empower 3 software (Waters) was used for data processing.

### Mass spectrometry of glycopeptides

Aliquots of 30–50 μg of recombinant HA proteins were denatured, reduced and alkylated as described previously [54]. For gel bands of virally derived HA, reduction and alkylation was performed according the protocol described by Shevchenko *et al*. [88]. Proteins were proteolytically digested with trypsin, chymotrypsin, and Glu-C (Promega). Reaction mixtures were lyophilized and peptides/glycopeptides were extracted using C18 Zip-tip (MerckMilipore) cleanup. Samples were resuspended in 0.1% formic acid then analyzed by liquid chromatography-mass spectrometry using an Easy-nLC 1200 system coupled to an Orbitrap Fusion mass spectrometer (Thermo Fisher Scientific). Glycopeptides were separated using an EasySpray PepMap RSLC C18 (75 μm × 75 cm) column with a 240-min linear solvent gradient of 0–32% acetonitrile in 0.1% formic acid, followed by 35 min of 80% acetonitrile in 0.1% formic acid. Mass spectrometry settings include an LC flow rate of 200 nL/min, spray voltage of 2.8 kV, capillary temperature of 275°C, and an HCD collision energy of 50%. Precursor and fragmentation detection were performed using an Orbitrap at the following resolution: MS1 = 100,000 and MS2 = 30,000. The automatic gain control (AGC) targets were MS1 = 4e^5^ and MS2 = 5e^4^, and injection times were MS1 = 50 and MS2 = 54. The following cleavage sites were used for the respective proteases; trypsin = R/K, chymotrypsin = F/Y/W, Glu C = E/D. The number of missed cleavages was set at 3. The following modifications were also included: Carbamidomethyl (+57.021464, target = C, fine control = fixed), Oxidation (+15.994915, target = M, fine control = variable rare 1), Glu to pyro-Glu (-18.010565, target = peptide N-term E, fine control = variable rare 1), and Gln to pyro-Glu (-17.026549, target peptide N-term Q, fine control = variable rare 1). Glycopeptide fragmentation data were extracted from raw files using Byonic (Version 3.5.0) and Byologic (Version 3.5–15; Protein Metrics Inc.). Glycopeptide fragmentation data were manually evaluated with true-positive assignments given when correct b- and y- fragments and oxonium ions corresponding to the peptide and glycan, respectively, were observed. The precursor mass tolerance was set at 4 ppm for precursor ions and 10 ppm for fragment ions. A 1% false discovery rate (FDR) was applied. The extracted ion chromatographic areas for each true-positive glycopeptide, with the same amino-acid sequence, were compared to determine the relative quantitation of glycoforms at each specific *N*-linked glycan site.

Fully glycosylated models of HK68 and Vic11 H3N2 HAs were created using crystal structures (PDB ID: 4FNK and 4O5N) [15, 89]. The most dominant glycoform presented at each site was modelled onto the *N*-linked carbohydrate sites in Coot [90].

## Supporting information

S1 Table Anti-carbohydrate HIV-1 antibodies analyzed in this study. (DOCX)

S2 Table Residue differences between Mos99 and Pan99 in the receptor binding domain. (DOCX)

S3 Table Human H3N2 viruses analyzed in this study. (DOCX)

S4 Table Primer sets for construction of Pan99 *N*-glycosylation HA library. (DOCX)

S1 Fig Neutralizing activity of 2G12, CR9114, and IgG311 antibodies against human H3N2 viruses. The 2G12 IgG only neutralized contemporary H3N2 influenza strains but not early ones. The anti-influenza virus broadly neutralizing antibody, CR9114 IgG, neutralized early and contemporary virus strains. The anti-malarial antibody, IgG311 showed no neutralizing activity against human H3N2 viruses. HK68, Bk79, SD93, Bris07, Vic11 were denoted by empty circles, filled circles, empty squares, filled squares, and empty diamonds, respectively. CR9114, 2G12, and IgG311 are represented in red, blue, and black. The IC_50_ > 400 μg/ml indicates no neutralizing activity.
(TIF)

S2 Fig Neutralizing activity of seven anti-carbohydrates antibodies against HIV-1 to human H3N2 viruses. Seven anti-carbohydrate antibodies against HIV-1 do not neutralize any H3N2 viruses at 200 μg/ml in a microneutralization assay. Dashed line represents the OD for healthy cell controls with no virus.
(TIF)

S3 Fig HILIC-UPLC *N*-linked glycan analysis. Chromatograms of fluorescently labelled *N*-linked glycans on HAs from H3N2 strains: HK68, Leningrad86, Shangdong93, Wyoming03, Perth09, Victoria11, and Michigan14. Note the dominant peak of Man_5_GlcNAc_2_ on HK68, whereas the 2G12 sensitive strains have prominent Man_8/9_GlcNAc_2_ peaks.
(TIF)

S4 Fig Site-specific *N*-linked glycosylation of viral and recombinant H3N2 HA glycoproteins. Viral and recombinant HAs from HK68 and Vic11 and their glycans analyzed by LC-MS. Glycans are colored according to the key.
(TIF)

S5 Fig Site-specific *N*-linked glycosylation of recombinant Bris07 wild type and N165A HA glycoproteins. Each N-glycan of the HAs was analyzed by LC-MS. Glycans are colored according to the key.
(TIF)

S6 Fig Binding kinetics and affinity of 2G12 to recombinant Bris07 WT and Bris07 N165A HA proteins. The binding of 2G12 IgG against recombinant Bris07 WT and Bris07 N165A HA proteins was measured by biolayer interferometry (BLI). Blue lines indicate the response curves and red dashed lines represent the fitting curve with 2:1 hetero-ligand binding model for both Bris07 WT and Bris07 N165A. Binding kinetics were measured ranging from 2000 nM to 250 nM by 2-fold serial dilution. The dissociation constant (*K*_off1_/ *K*_off2_), association constant (*K*_on1_/ *K*_on2_), equilibrium dissociation constant (*K*_d1_/ *K*_d2_), and R^2^ of the fitting are summarized.
(TIF)

S7 Fig The spatial distribution of *N*-glycosylation sites involved in 2G12 recognition on HIV-1 Env trimer and influenza H3 hemagglutinin. **(A)** Front view of HIV-1 Env trimer (PDB: 4ZMJ) [97]. Oligomannose on four *N*-glycosylation sites, N295 (red), N332 (marine), N339 (yellow), and N392 (purple) are involved in 2G12 recognition [11,46]. **(B)** Close-up view of N295 and N332 on HIV-1 Env. The distance between the Cα’s of N295 and N332 is 4.6Å (yellow dashed line). **(C)** Top view of influenza H3 hemagglutinin trimer (PDB: 6AOV) [91]. Oligomannose on two *N*-glycosylation sites, N165 (red) and N246 (marine), are involved in recognition by 2G12. **(D)** Close-up view of H3 hemagglutinin trimer around N165 and N246. The distance between Cα of N165 and N246 on adjacent strands of a beta-sheet is also 4.6Å (yellow dashed line).
(TIF)

S8 Fig Glyco-network of N165 and N246 on HA. Schematic of Man_9_GlcNAc_2_ (left panel). The D1, D2, and D3 arms of Man_9_GlcNAc_2_ are in black, yellow, and purple, respectively, and GlcNAc_2_ in green here and in the other panels. Top view of A/Brisbane/10/07 (H3N2) HA (PDB 6AOV) [91]. Glycans on N165 and N246 were modelled with Man_9_GlcNAc_2_ by Charmm-Gui [92] (middle panel). The location of N165 and N246 in each protomer is in red and cyan, respectively. Distances between the D3 arm of N246 to the D1 and D3 arms of N165 are 26 Å and 29 Å, respectively in the same protomer. The right panel illustrates triangles formed by D1 arms (black triangle) and D3 arms (purple triangle) of Man_9_GlcNAc_2_ on each protomer of HA. The distances between the ends of D1 to D1, and D3 to D3 of two N246 on different HA protomers are 14 Å and 44 Å, respectively.
(TIF)

## References

[1] Trkola A, Purtscher M, Muster T, Ballaun C, Buchacher A, Sullivan N, et al. Human monoclonal antibody 2G12 defines a distinctive neutralization epitope on the gp120 glycoprotein of human immunodeficiency virus type 1. J Virol. 1996;70(2):1100–8. Epub 1996/02/01. 10.1128/JVI.70.2.1100-1108.1996 ; PubMed Central PMCID: 189917. 8551569 PMC189917

[2] Buchacher A, Predl R, Strutzenberger K, Steinfellner W, Trkola A, Purtscher M, et al. Generation of human monoclonal antibodies against HIV-1 proteins; electrofusion and Epstein-Barr virus transformation for peripheral blood lymphocyte immortalization. AIDS Res Hum Retroviruses. 1994;10(4):359–69. Epub 1994/04/01. 10.1089/aid.1994.10.359 . 7520721

[3] Walker LM, Phogat SK, Chan-Hui PY, Wagner D, Phung P, Goss JL, et al. Broad and potent neutralizing antibodies from an African donor reveal a new HIV-1 vaccine target. Science. 2009;326(5950):285–9. Epub 2009/09/05. 10.1126/science.1178746 ; PubMed Central PMCID: 3335270. 19729618 PMC3335270

[4] Walker LM, Huber M, Doores KJ, Falkowska E, Pejchal R, Julien JP, et al. Broad neutralization coverage of HIV by multiple highly potent antibodies. Nature. 2011;477(7365):466–70. Epub 2011/08/19. 10.1038/nature10373 ; PubMed Central PMCID: 3393110. 21849977 PMC3393110

[5] Wu X, Yang ZY, Li Y, Hogerkorp CM, Schief WR, Seaman MS, et al. Rational design of envelope identifies broadly neutralizing human monoclonal antibodies to HIV-1. Science. 2010;329(5993):856–61. 10.1126/science.1187659 ; PubMed Central PMCID: 2965066. 20616233 PMC2965066

[6] Sok D, van Gils MJ, Pauthner M, Julien JP, Saye-Francisco KL, Hsueh J, et al. Recombinant HIV envelope trimer selects for quaternary-dependent antibodies targeting the trimer apex. Proc Natl Acad Sci U S A. 2014;111(49):17624–9. Epub 2014/11/26. 10.1073/pnas.1415789111 ; PubMed Central PMCID: 4267403. 25422458 PMC4267403

[7] Mouquet H, Scharf L, Euler Z, Liu Y, Eden C, Scheid JF, et al. Complex-type N-glycan recognition by potent broadly neutralizing HIV antibodies. Proc Natl Acad Sci U S A. 2012;109(47):E3268–77. Epub 2012/11/02. 10.1073/pnas.1217207109 ; PubMed Central PMCID: 3511153. 23115339 PMC3511153

[8] Burton DR, Hangartner L. Broadly neutralizing antibodies to HIV and their role in vaccine design. Annu Rev Immunol. 2016;34:635–59. Epub 2016/05/12. 10.1146/annurev-immunol-041015-055515 ; PubMed Central PMCID: 6034635. 27168247 PMC6034635

[9] Sok D, Burton DR. Recent progress in broadly neutralizing antibodies to HIV. Nat Immunol. 2018;19(11):1179–88. Epub 2018/10/20. 10.1038/s41590-018-0235-7 ; PubMed Central PMCID: 6440471. 30333615 PMC6440471

[10] Burton DR, Ahmed R, Barouch DH, Butera ST, Crotty S, Godzik A, et al. A blueprint for HIV vaccine discovery. Cell Host Microbe. 2012;12(4):396–407. Epub 2012/10/23. 10.1016/j.chom.2012.09.008 ; PubMed Central PMCID: 3513329. 23084910 PMC3513329

[11] Calarese DA, Scanlan CN, Zwick MB, Deechongkit S, Mimura Y, Kunert R, et al. Antibody domain exchange is an immunological solution to carbohydrate cluster recognition. Science. 2003;300(5628):2065–71. Epub 2003/06/28. 10.1126/science.1083182 . 12829775

[12] Pejchal R, Doores KJ, Walker LM, Khayat R, Huang PS, Wang SK, et al. A potent and broad neutralizing antibody recognizes and penetrates the HIV glycan shield. Science. 2011;334(6059):1097–103. Epub 2011/10/15. 10.1126/science.1213256 ; PubMed Central PMCID: 3280215. 21998254 PMC3280215

[13] Pinto D, Park YJ, Beltramello M, Walls AC, Tortorici MA, Bianchi S, et al. Cross-neutralization of SARS-CoV-2 by a human monoclonal SARS-CoV antibody. Nature. 2020. Epub 2020/05/19. 10.1038/s41586-020-2349-y . 32422645

[14] Rouvinski A, Guardado-Calvo P, Barba-Spaeth G, Duquerroy S, Vaney MC, Kikuti CM, et al. Recognition determinants of broadly neutralizing human antibodies against dengue viruses. Nature. 2015;520(7545):109–13. Epub 2015/01/13. 10.1038/nature14130 . 25581790

[15] Lee PS, Ohshima N, Stanfield RL, Yu W, Iba Y, Okuno Y, et al. Receptor mimicry by antibody F045-092 facilitates universal binding to the H3 subtype of influenza virus. Nat Commun. 2014;5:3614. 10.1038/ncomms4614 ; PubMed Central PMCID: 4358779. 24717798 PMC4358779

[16] Abe Y, Takashita E, Sugawara K, Matsuzaki Y, Muraki Y, Hongo S. Effect of the addition of oligosaccharides on the biological activities and antigenicity of influenza A/H3N2 virus hemagglutinin. J Virol. 2004;78(18):9605–11. 10.1128/JVI.78.18.9605-9611.2004 ; PubMed Central PMCID: 514993. 15331693 PMC514993

[17] Wu NC, Wilson IA. A perspective on the structural and functional constraints for immune evasion: insights from influenza virus. J Mol Biol. 2017;429(17):2694–709. 10.1016/j.jmb.2017.06.015 ; PubMed Central PMCID: 5573227. 28648617 PMC5573227

[18] Altman MO, Angel M, Kosik I, Trovao NS, Zost SJ, Gibbs JS, et al. Human influenza A virus hemagglutinin glycan evolution follows a temporal pattern to a glycan limit. mBio. 2019;10(2). Epub 2019/04/04. 10.1128/mBio.00204-19 ; PubMed Central PMCID: 6445938. 30940704 PMC6445938

[19] Igarashi M, Ito K, Kida H, Takada A. Genetically destined potentials for N-linked glycosylation of influenza virus hemagglutinin. Virology. 2008;376(2):323–9. 10.1016/j.virol.2008.03.036 . 18456302

[20] Skehel JJ, Stevens DJ, Daniels RS, Douglas AR, Knossow M, Wilson IA, et al. A carbohydrate side chain on hemagglutinins of Hong Kong influenza viruses inhibits recognition by a monoclonal antibody. Proc Natl Acad Sci U S A. 1984;81(6):1779–83. 10.1073/pnas.81.6.1779 ; PubMed Central PMCID: 345004. 6584912 PMC345004

[21] Anders EM, Hartley CA, Reading PC, Ezekowitz RA. Complement-dependent neutralization of influenza virus by a serum mannose-binding lectin. J Gen Virol. 1994;75 (Pt 3):615–22. Epub 1994/03/01. 10.1099/0022-1317-75-3-615 . 8126457

[22] Hartshorn KL, Sastry K, White MR, Anders EM, Super M, Ezekowitz RA, et al. Human mannose-binding protein functions as an opsonin for influenza A viruses. J Clin Invest. 1993;91(4):1414–20. Epub 1993/04/01. 10.1172/JCI116345 ; PubMed Central PMCID: 288115. 7682571 PMC288115

[23] Kase T, Suzuki Y, Kawai T, Sakamoto T, Ohtani K, Eda S, et al. Human mannan-binding lectin inhibits the infection of influenza A virus without complement. Immunology. 1999;97(3):385–92. Epub 1999/08/14. 10.1046/j.1365-2567.1999.00781.x ; PubMed Central PMCID: 2326860. 10447758 PMC2326860

[24] Chang WC, White MR, Moyo P, McClear S, Thiel S, Hartshorn KL, et al. Lack of the pattern recognition molecule mannose-binding lectin increases susceptibility to influenza A virus infection. BMC Immunol. 2010;11:64. Epub 2010/12/25. 10.1186/1471-2172-11-64 ; PubMed Central PMCID: 3022599. 21182784 PMC3022599

[25] Garces F, Sok D, Kong L, McBride R, Kim HJ, Saye-Francisco KF, et al. Structural evolution of glycan recognition by a family of potent HIV antibodies. Cell. 2014;159(1):69–79. Epub 2014/09/27. 10.1016/j.cell.2014.09.009 ; PubMed Central PMCID: 4278586. 25259921 PMC4278586

[26] Scanlan CN, Pantophlet R, Wormald MR, Ollmann Saphire E, Stanfield R, Wilson IA, et al. The broadly neutralizing anti-human immunodeficiency virus type 1 antibody 2G12 recognizes a cluster of α1→2 mannose residues on the outer face of gp120. J Virol. 2002;76(14):7306–21. Epub 2002/06/20. 10.1128/jvi.76.14.7306-7321.2002 ; PubMed Central PMCID: 136327. 12072529 PMC136327

[27] Seabright GE, Cottrell CA, van Gils MJ, D’Addabbo A, Harvey DJ, Behrens AJ, et al. Networks of HIV-1 envelope glycans maintain antibody epitopes in the face of glycan additions and deletions. Structure. 2020;28(8):897–909.e6. Epub 2020/05/21. 10.1016/j.str.2020.04.022 . 32433992 PMC7416112

[28] Murin CD, Julien JP, Sok D, Stanfield RL, Khayat R, Cupo A, et al. Structure of 2G12 Fab_2_ in complex with soluble and fully glycosylated HIV-1 Env by negative-stain single-particle electron microscopy. J Virol. 2014;88(17):10177–88. Epub 2014/06/27. 10.1128/JVI.01229-14 ; PubMed Central PMCID: 4136306. 24965454 PMC4136306

[29] Wilson IA, Skehel JJ, Wiley DC. Structure of the haemagglutinin membrane glycoprotein of influenza virus at 3 Å resolution. Nature. 1981;289(5796):366–73. 10.1038/289366a0 . 7464906

[30] Dreyfus C, Laursen NS, Kwaks T, Zuijdgeest D, Khayat R, Ekiert DC, et al. Highly conserved protective epitopes on influenza B viruses. Science. 2012;337(6100):1343–8. Epub 2012/08/11. 10.1126/science.1222908 ; PubMed Central PMCID: 3538841. 22878502 PMC3538841

[31] Oyen D, Torres JL, Cottrell CA, Richter King C, Wilson IA, Ward AB. Cryo-EM structure of *P*. *falciparum* circumsporozoite protein with a vaccine-elicited antibody is stabilized by somatically mutated inter-Fab contacts. Sci Adv. 2018;4(10):eaau8529. Epub 2018/10/17. 10.1126/sciadv.aau8529 ; PubMed Central PMCID: 6179375. 30324137 PMC6179375

[32] Georgiev IS, Doria-Rose NA, Zhou T, Kwon YD, Staupe RP, Moquin S, et al. Delineating antibody recognition in polyclonal sera from patterns of HIV-1 isolate neutralization. Science. 2013;340(6133):751–6. Epub 2013/05/11. 10.1126/science.1233989 . 23661761

[33] Lee JH, Andrabi R, Su CY, Yasmeen A, Julien JP, Kong L, et al. A broadly neutralizing antibody targets the dynamic HIV envelope trimer apex via a long, rigidified, and anionic beta-hairpin structure. Immunity. 2017;46(4):690–702. Epub 2017/04/20. 10.1016/j.immuni.2017.03.017 ; PubMed Central PMCID: 5400778. 28423342 PMC5400778

[34] Barnes CO, Gristick HB, Freund NT, Escolano A, Lyubimov AY, Hartweger H, et al. Structural characterization of a highly-potent V3-glycan broadly neutralizing antibody bound to natively-glycosylated HIV-1 envelope. Nat Commun. 2018;9(1):1251. Epub 2018/03/30. 10.1038/s41467-018-03632-y ; PubMed Central PMCID: 5871869. 29593217 PMC5871869

[35] Huang J, Kang BH, Pancera M, Lee JH, Tong T, Feng Y, et al. Broad and potent HIV-1 neutralization by a human antibody that binds the gp41-gp120 interface. Nature. 2014;515(7525):138–42. Epub 2014/09/05. 10.1038/nature13601 ; PubMed Central PMCID: 4224615. 25186731 PMC4224615

[36] Julien JP, Cupo A, Sok D, Stanfield RL, Lyumkis D, Deller MC, et al. Crystal structure of a soluble cleaved HIV-1 envelope trimer. Science. 2013;342(6165):1477–83. Epub 2013/11/02. 10.1126/science.1245625 ; PubMed Central PMCID: 3886632. 24179159 PMC3886632

[37] Freund NT, Wang H, Scharf L, Nogueira L, Horwitz JA, Bar-On Y, et al. Coexistence of potent HIV-1 broadly neutralizing antibodies and antibody-sensitive viruses in a viremic controller. Sci Transl Med. 2017;9(373). Epub 2017/01/20. 10.1126/scitranslmed.aal2144 ; PubMed Central PMCID: 5467220. 28100831 PMC5467220

[38] An Y, McCullers JA, Alymova I, Parsons LM, Cipollo JF. Glycosylation analysis of engineered H3N2 influenza A virus hemagglutinins with sequentially added historically relevant glycosylation sites. J Proteome Res. 2015;14(9):3957–69. Epub 2015/07/24. 10.1021/acs.jproteome.5b00416 . 26202417

[39] An Y, Parsons LM, Jankowska E, Melnyk D, Joshi M, Cipollo JF. N-glycosylation of seasonal influenza vaccine hemagglutinins: implication for potency testing and immune processing. J Virol. 2019;93(2):e01693–18. Epub 2018/10/26. 10.1128/JVI.01693-18 ; PubMed Central PMCID: 6321900. 30355697 PMC6321900

[40] Khatri K, Klein JA, White MR, Grant OC, Leymarie N, Woods RJ, et al. Integrated omics and computational glycobiology reveal structural basis for influenza A virus glycan microheterogeneity and host interactions. Mol Cell Proteomics. 2016;15(6):1895–912. Epub 2016/03/18. 10.1074/mcp.M116.058016 ; PubMed Central PMCID: 5083086. 26984886 PMC5083086

[41] Ha Y, Stevens DJ, Skehel JJ, Wiley DC. H5 avian and H9 swine influenza virus haemagglutinin structures: possible origin of influenza subtypes. EMBO J. 2002;21(5):865–75. 10.1093/emboj/21.5.865 ; PubMed Central PMCID: 125880. 11867515 PMC125880

[42] Vigerust DJ, Ulett KB, Boyd KL, Madsen J, Hawgood S, McCullers JA. N-linked glycosylation attenuates H3N2 influenza viruses. J Virol. 2007;81(16):8593–600. Epub 2007/06/08. 10.1128/JVI.00769-07 ; PubMed Central PMCID: 1951338. 17553891 PMC1951338

[43] Pritchard LK, Spencer DI, Royle L, Bonomelli C, Seabright GE, Behrens AJ, et al. Glycan clustering stabilizes the mannose patch of HIV-1 and preserves vulnerability to broadly neutralizing antibodies. Nat Commun. 2015;6:7479. Epub 2015/06/25. 10.1038/ncomms8479 ; PubMed Central PMCID: 4500839. 26105115 PMC4500839

[44] Watanabe Y, Raghwani J, Allen JD, Seabright GE, Li S, Moser F, et al. Structure of the Lassa virus glycan shield provides a model for immunological resistance. Proc Natl Acad Sci U S A. 2018;115(28):7320–5. Epub 2018/06/27. 10.1073/pnas.1803990115 ; PubMed Central PMCID: 6048489. 29941589 PMC6048489

[45] Watanabe Y, Berndsen ZT, Raghwani J, Seabright GE, Allen JD, Pybus OG, et al. Vulnerabilities in coronavirus glycan shields despite extensive glycosylation. Nat Commun. 2020;11(1):2688. Epub 2020/05/29. 10.1038/s41467-020-16567-0 ; PubMed Central PMCID: 7253482. 32461612 PMC7253482

[46] Seabright GE, Doores KJ, Burton DR, Crispin M. Protein and glycan mimicry in HIV vaccine design. J Mol Biol. 2019;431(12):2223–47. Epub 2019/04/28. 10.1016/j.jmb.2019.04.016 ; PubMed Central PMCID: 6556556. 31028779 PMC6556556

[47] Scanlan CN, Pantophlet R, Wormald MR, Saphire EO, Calarese D, Stanfield R, et al. The carbohydrate epitope of the neutralizing anti-HIV-1 antibody 2G12. Adv Exp Med Biol. 2003;535:205–18. Epub 2004/01/13. 10.1007/978-1-4615-0065-0_13 . 14714897

[48] Song R, Oren DA, Franco D, Seaman MS, Ho DD. Strategic addition of an N-linked glycan to a monoclonal antibody improves its HIV-1-neutralizing activity. Nat Biotechnol. 2013;31(11):1047–52. Epub 2013/10/08. 10.1038/nbt.2677 ; PubMed Central PMCID: 3825789. 24097413 PMC3825789

[49] Francica JR, Varela-Rohena A, Medvec A, Plesa G, Riley JL, Bates P. Steric shielding of surface epitopes and impaired immune recognition induced by the ebola virus glycoprotein. PLoS Pathog. 2010;6(9):e1001098. Epub 2010/09/17. 10.1371/journal.ppat.1001098 ; PubMed Central PMCID: 2936550. 20844579 PMC2936550

[50] Watanabe Y, Bowden TA, Wilson IA, Crispin M. Exploitation of glycosylation in enveloped virus pathobiology. Biochim Biophys Acta Gen Subj. 2019;1863(10):1480–97. Epub 2019/05/24. 10.1016/j.bbagen.2019.05.012 ; PubMed Central PMCID: 6686077. 31121217 PMC6686077

[51] Dey AK, Cupo A, Ozorowski G, Sharma VK, Behrens AJ, Go EP, et al. cGMP production and analysis of BG505 SOSIP.664, an extensively glycosylated, trimeric HIV-1 envelope glycoprotein vaccine candidate. Biotechnol Bioeng. 2018;115(4):885–99. Epub 2017/11/19. 10.1002/bit.26498 ; PubMed Central PMCID: 5852640. 29150937 PMC5852640

[52] Cao L, Pauthner M, Andrabi R, Rantalainen K, Berndsen Z, Diedrich JK, et al. Differential processing of HIV envelope glycans on the virus and soluble recombinant trimer. Nat Commun. 2018;9(1):3693. Epub 2018/09/14. 10.1038/s41467-018-06121-4 ; PubMed Central PMCID: 6135743. 30209313 PMC6135743

[53] Struwe WB, Chertova E, Allen JD, Seabright GE, Watanabe Y, Harvey DJ, et al. Site-specific glycosylation of virion-derived HIV-1 Env is mimicked by a soluble trimeric immunogen. Cell Rep. 2018;24(8):1958–66 e5. Epub 2018/08/23. 10.1016/j.celrep.2018.07.080 ; PubMed Central PMCID: 6113929. 30134158 PMC6113929

[54] Watanabe Y, Allen JD, Wrapp D, McLellan JS, Crispin M. Site-specific glycan analysis of the SARS-CoV-2 spike. Science. 2020;369(6501):330–3. Epub 2020/05/06. 10.1126/science.abb9983 ; PubMed Central PMCID: 7199903. 32366695 PMC7199903

[55] Crispin M, Ward AB, Wilson IA. Structure and immune recognition of the HIV glycan shield. Annu Rev Biophys. 2018;47:499–523. Epub 2018/03/30. 10.1146/annurev-biophys-060414-034156 ; PubMed Central PMCID: 6163090. 29595997 PMC6163090

[56] Kong L, Lee JH, Doores KJ, Murin CD, Julien JP, McBride R, et al. Supersite of immune vulnerability on the glycosylated face of HIV-1 envelope glycoprotein gp120. Nat Struct Mol Biol. 2013;20(7):796–803. Epub 2013/05/28. 10.1038/nsmb.2594 ; PubMed Central PMCID: 3823233. 23708606 PMC3823233

[57] Webb NE, Montefiori DC, Lee B. Dose-response curve slope helps predict therapeutic potency and breadth of HIV broadly neutralizing antibodies. Nat Commun. 2015;6:8443. Epub 2015/09/30. 10.1038/ncomms9443 ; PubMed Central PMCID: 4588098. 26416571 PMC4588098

[58] Calarese DA, Lee HK, Huang CY, Best MD, Astronomo RD, Stanfield RL, et al. Dissection of the carbohydrate specificity of the broadly neutralizing anti-HIV-1 antibody 2G12. Proc Natl Acad Sci U S A. 2005;102(38):13372–7. Epub 2005/09/22. 10.1073/pnas.0505763102 ; PubMed Central PMCID: 1224641. 16174734 PMC1224641

[59] Hou W, Fang C, Liu J, Yu H, Qi J, Zhang Z, et al. Molecular insights into the inhibition of HIV-1 infection using a CD4 domain-1-specific monoclonal antibody. Antiviral Res. 2015;122:101–11. Epub 2015/08/12. 10.1016/j.antiviral.2015.08.004 . 26259811

[60] Zhang L, Irimia A, He L, Landais E, Rantalainen K, Leaman DP, et al. An MPER antibody neutralizes HIV-1 using germline features shared among donors. Nat Commun. 2019;10(1):5389. Epub 2019/11/28. 10.1038/s41467-019-12973-1 ; PubMed Central PMCID: 6879610. 31772165 PMC6879610

[61] Wu X, Guo J, Niu M, An M, Liu L, Wang H, et al. Tandem bispecific neutralizing antibody eliminates HIV-1 infection in humanized mice. J Clin Invest. 2018;128(6):2239–51. Epub 2018/02/21. 10.1172/JCI96764 ; PubMed Central PMCID: 5983313. 29461979 PMC5983313

[62] Bayer K, Banning C, Bruss V, Wiltzer-Bach L, Schindler M. Hepatitis C virus is released via a noncanonical secretory route. J Virol. 2016;90(23):10558–73. Epub 2016/09/16. 10.1128/JVI.01615-16 ; PubMed Central PMCID: 5110177. 27630244 PMC5110177

[63] Powlesland AS, Fisch T, Taylor ME, Smith DF, Tissot B, Dell A, et al. A novel mechanism for LSECtin binding to Ebola virus surface glycoprotein through truncated glycans. J Biol Chem. 2008;283(1):593–602. Epub 2007/11/07. 10.1074/jbc.M706292200 ; PubMed Central PMCID: 2275798. 17984090 PMC2275798

[64] Acharya P, Williams W, Henderson R, Janowska K, Manne K, Parks R, et al. A glycan cluster on the SARS-CoV-2 spike ectodomain is recognized by Fab-dimerized glycan-reactive antibodies. bioRxiv. 2020:2020.06.30.178897. 10.1101/2020.06.30.178897 32637953 PMC7337383

[65] Simmons G, Reeves JD, Grogan CC, Vandenberghe LH, Baribaud F, Whitbeck JC, et al. DC-SIGN and DC-SIGNR bind ebola glycoproteins and enhance infection of macrophages and endothelial cells. Virology. 2003;305(1):115–23. Epub 2002/12/31. 10.1006/viro.2002.1730 . 12504546

[66] Mason CP, Tarr AW. Human lectins and their roles in viral infections. Molecules. 2015;20(2):2229–71. Epub 2015/02/03. 10.3390/molecules20022229 ; PubMed Central PMCID: 6272597. 25642836 PMC6272597

[67] Thompson AJ, Cao L, Ma Y, Wang X, Diedrich JK, Kikuchi C, et al. Human influenza virus hemagglutinins contain conserved oligomannose N-linked glycans allowing potent neutralization by lectins. Cell Host Microbe. 2020;27(5):725–35 e5. Epub 2020/04/17. 10.1016/j.chom.2020.03.009 ; PubMed Central PMCID: 7158820. 32298658 PMC7158820

[68] Williams WB, Meyerhoff RR, Edwards R, Li H, Nicely NI, Henderson R, et al. Fab-dimerized glycan-reactive antibodies neutralize HIV and are prevalent in humans and rhesus macaques. bioRxiv. 2020:2020.06.30.178921. 10.1101/2020.06.30.178921

[69] Katoh K, Misawa K, Kuma K, Miyata T. MAFFT: a novel method for rapid multiple sequence alignment based on fast Fourier transform. Nucleic Acids Res. 2002;30(14):3059–66. Epub 2002/07/24. 10.1093/nar/gkf436 ; PubMed Central PMCID: 135756. 12136088 PMC135756

[70] R Core Team. R: A language and environment for statistical computing.: R Foundation for Statistical Computing; 2018 [cited 2020]. Available from: https://www.R-project.org/.

[71] Schrodinger LLC. The PyMOL Molecular Graphics System, Version 1.8. 2015.

[72] Wu NC, Otwinowski J, Thompson AJ, Nycholat CM, Nourmohammad A, Wilson IA. Major antigenic site B of human influenza H3N2 viruses has an evolving local fitness landscape. Nat Commun. 2020;11(1):1233. Epub 2020/03/08. 10.1038/s41467-020-15102-5 ; PubMed Central PMCID: 7060233. 32144244 PMC7060233

[73] Yang JR, Hsu SZ, Kuo CY, Huang HY, Huang TY, Wang HC, et al. An epidemic surge of influenza A(H3N2) virus at the end of the 2016–2017 season in Taiwan with an increased viral genetic heterogeneity. J Clin Virol. 2018;99–100:15–21. Epub 2017/12/27. 10.1016/j.jcv.2017.12.012 . 29278832

[74] Tsou TP, Su CP, Huang WT, Yang JR, Liu MT. Influenza A(H3N2) virus variants and patient characteristics during a summer influenza epidemic in Taiwan, 2017. Euro Surveill. 2017;22(50):17–00767. Epub 2017/12/21. 10.2807/1560-7917.ES.2017.22.50.17–00767 ; PubMed Central PMCID: 5743095. 29258649 PMC5743095

[75] Reed LJ, Muench H. A simple method of estimating fifty percent endpoints. Am J Epidem. 1938;27(3):493–7. 10.1093/oxfordjournals.aje.a118408

[76] Wu NC, Grande G, Turner HL, Ward AB, Xie J, Lerner RA, et al. In vitro evolution of an influenza broadly neutralizing antibody is modulated by hemagglutinin receptor specificity. Nat Commun. 2017;8:15371. 10.1038/ncomms15371 ; PubMed Central PMCID: 5440694. 28504265 PMC5440694

[77] Joyce MG, Wheatley AK, Thomas PV, Chuang GY, Soto C, Bailer RT, et al. Vaccine-induced antibodies that neutralize group 1 and group 2 influenza A viruses. Cell. 2016;166(3):609–23. Epub 2016/07/28. 10.1016/j.cell.2016.06.043 ; PubMed Central PMCID: 4978566. 27453470 PMC4978566

[78] Ekiert DC, Friesen RH, Bhabha G, Kwaks T, Jongeneelen M, Yu W, et al. A highly conserved neutralizing epitope on group 2 influenza A viruses. Science. 2011;333(6044):843–50. 10.1126/science.1204839 ; PubMed Central PMCID: 3210727. 21737702 PMC3210727

[79] Peng W, de Vries RP, Grant OC, Thompson AJ, McBride R, Tsogtbaatar B, et al. Recent H3N2 viruses have evolved specificity for extended, branched human-type receptors, conferring potential for increased avidity. Cell Host Microbe. 2017;21(1):23–34. 10.1016/j.chom.2016.11.004 ; PubMed Central PMCID: 5233592. 28017661 PMC5233592

[80] Wu NC, Thompson AJ, Xie J, Lin CW, Nycholat CM, Zhu X, et al. A complex epistatic network limits the mutational reversibility in the influenza hemagglutinin receptor-binding site. Nat Commun. 2018;9(1):1264. 10.1038/s41467-018-03663-5 ; PubMed Central PMCID: 5871881. 29593268 PMC5871881

[81] Crooks GE, Hon G, Chandonia JM, Brenner SE. WebLogo: a sequence logo generator. Genome Res. 2004;14(6):1188–90. Epub 2004/06/03. 10.1101/gr.849004 ; PubMed Central PMCID: 419797. 15173120 PMC419797

[82] Suloway C, Pulokas J, Fellmann D, Cheng A, Guerra F, Quispe J, et al. Automated molecular microscopy: the new Leginon system. J Struct Biol. 2005;151(1):41–60. Epub 2005/05/14. 10.1016/j.jsb.2005.03.010 . 15890530

[83] Lander GC, Stagg SM, Voss NR, Cheng A, Fellmann D, Pulokas J, et al. Appion: an integrated, database-driven pipeline to facilitate EM image processing. J Struct Biol. 2009;166(1):95–102. 10.1016/j.jsb.2009.01.002 ; PubMed Central PMCID: 2775544. 19263523 PMC2775544

[84] Zivanov J, Nakane T, Forsberg BO, Kimanius D, Hagen WJ, Lindahl E, et al. New tools for automated high-resolution cryo-EM structure determination in RELION-3. eLife. 2018;7:e42166. Epub 2018/11/10. 10.7554/eLife.42166 ; PubMed Central PMCID: 6250425. 30412051 PMC6250425

[85] Scheres SH. RELION: implementation of a Bayesian approach to cryo-EM structure determination. J Struct Biol. 2012;180(3):519–30. Epub 2012/09/25. 10.1016/j.jsb.2012.09.006 ; PubMed Central PMCID: 3690530. 23000701 PMC3690530

[86] Zost SJ, Lee J, Gumina ME, Parkhouse K, Henry C, Wu NC, et al. Identification of antibodies targeting the H3N2 hemagglutinin receptor binding site following vaccination of humans. Cell Rep. 2019;29(13):4460–70 e8. Epub 2019/12/26. 10.1016/j.celrep.2019.11.084 ; PubMed Central PMCID: 6953393. 31875553 PMC6953393

[87] Wu NC, Xie J, Zheng T, Nycholat CM, Grande G, Paulson JC, et al. Diversity of functionally permissive sequences in the receptor-binding site of influenza hemagglutinin. Cell Host Microbe. 2017;21(6):742–53 e8. Epub 2017/06/16. 10.1016/j.chom.2017.05.011 ; PubMed Central PMCID: 5553561. 28618270 PMC5553561

[88] Shevchenko A, Tomas H, Havlis J, Olsen JV, Mann M. In-gel digestion for mass spectrometric characterization of proteins and proteomes. Nat Protoc. 2006;1(6):2856–60. Epub 2007/04/05. 10.1038/nprot.2006.468 . 17406544

[89] Ekiert DC, Kashyap AK, Steel J, Rubrum A, Bhabha G, Khayat R, et al. Cross-neutralization of influenza A viruses mediated by a single antibody loop. Nature. 2012;489(7417):526–32. Epub 2012/09/18. 10.1038/nature11414 ; PubMed Central PMCID: 3538848. 22982990 PMC3538848

[90] Emsley P, Crispin M. Structural analysis of glycoproteins: building N-linked glycans with Coot. Acta Crystallogr D Struct Biol. 2018;74(Pt 4):256–63. Epub 2018/04/14. 10.1107/S2059798318005119 ; PubMed Central PMCID: 5892875. 29652253 PMC5892875

[91] Wu NC, Zost SJ, Thompson AJ, Oyen D, Nycholat CM, McBride R, et al. A structural explanation for the low effectiveness of the seasonal influenza H3N2 vaccine. PLoS Pathog. 2017;13(10):e1006682. 10.1371/journal.ppat.1006682 . 29059230 PMC5667890

[92] Jo S, Kim T, Iyer VG, Im W. CHARMM-GUI: a web-based graphical user interface for CHARMM. J Comput Chem. 2008;29(11):1859–65. Epub 2008/03/21. 10.1002/jcc.20945 . 18351591

[93] Yuan Y, Cao D, Zhang Y, Ma J, Qi J, Wang Q, et al. Cryo-EM structures of MERS-CoV and SARS-CoV spike glycoproteins reveal the dynamic receptor binding domains. Nat Commun. 2017;8:15092. Epub 2017/04/11. 10.1038/ncomms15092 ; PubMed Central PMCID: 5394239. 28393837 PMC5394239

[94] Wrapp D, Wang N, Corbett KS, Goldsmith JA, Hsieh CL, Abiona O, et al. Cryo-EM structure of the 2019-nCoV spike in the prefusion conformation. Science. 2020;367(6483):1260–3. Epub 2020/02/23. 10.1126/science.abb2507 ; PubMed Central PMCID: 7164637. 32075877 PMC7164637

[95] Hastie KM, Zandonatti MA, Kleinfelter LM, Heinrich ML, Rowland MM, Chandran K, et al. Structural basis for antibody-mediated neutralization of Lassa virus. Science. 2017;356(6341):923–8. Epub 2017/06/03. 10.1126/science.aam7260 ; PubMed Central PMCID: 6007842. 28572385 PMC6007842

[96] Lee JH, de Val N, Lyumkis D, Ward AB. Model building and refinement of a natively glycosylated HIV-1 Env protein by high-resolution cryoelectron microscopy. Structure. 2015;23(10):1943–51. Epub 2015/09/22. 10.1016/j.str.2015.07.020 ; PubMed Central PMCID: 4618500. 26388028 PMC4618500

[97] Kwon YD, Pancera M, Acharya P, Georgiev IS, Crooks ET, Gorman J, et al. Crystal structure, conformational fixation and entry-related interactions of mature ligand-free HIV-1 Env. Nat Struct Mol Biol. 2015;22(7):522–31. Epub 2015/06/23. 10.1038/nsmb.3051 ; PubMed Central PMCID: 4706170. 26098315 PMC4706170

