# Supplementary material for: A cross-neutralizing antibody between HIV-1 and influenza virus
Source: PLoS Pathog. 2021 Mar 22;17(3):e1009407. doi: 10.1371/journal.ppat.1009407 (PMC8016226; doi:10.1371/journal.ppat.1009407)
Supplement: S1 Table — (DOCX) [file ppat.1009407.s001.docx]

| Name | Reference |
| --- | --- |
| 2G12 | [1-3] |
| PGT128 | [4,5] |
| VRC24 | [6] |
| PGT145 | [4,5] |
| BG18 | [7] |
| PGDM1400 | [8] |
| 35O22 | [9] |
| PGT135 | [4,5] |
| CR9114 | [10] |
| IgG311 | [11] |

**Reference**

1. Trkola A, Purtscher M, Muster T, Ballaun C, Buchacher A, Sullivan N, et al. Human monoclonal antibody 2G12 defines a distinctive neutralization epitope on the gp120 glycoprotein of human immunodeficiency virus type 1. J Virol. 1996;70(2):1100-8. Epub 1996/02/01. PubMed PMID: 8551569; PubMed Central PMCID: 189917.

2. Buchacher A, Predl R, Strutzenberger K, Steinfellner W, Trkola A, Purtscher M, et al. Generation of human monoclonal antibodies against HIV-1 proteins; electrofusion and Epstein-Barr virus transformation for peripheral blood lymphocyte immortalization. AIDS Res Hum Retroviruses. 1994;10(4):359-69. Epub 1994/04/01. doi: 10.1089/aid.1994.10.359. PubMed PMID: 7520721.

3. Calarese DA, Scanlan CN, Zwick MB, Deechongkit S, Mimura Y, Kunert R, et al. Antibody domain exchange is an immunological solution to carbohydrate cluster recognition. Science. 2003;300(5628):2065-71. Epub 2003/06/28. doi: 10.1126/science.1083182. PubMed PMID: 12829775.

4. Walker LM, Huber M, Doores KJ, Falkowska E, Pejchal R, Julien JP, et al. Broad neutralization coverage of HIV by multiple highly potent antibodies. Nature. 2011;477(7365):466-70. Epub 2011/08/19. doi: 10.1038/nature10373. PubMed PMID: 21849977; PubMed Central PMCID: 3393110.

5. Julien JP, Cupo A, Sok D, Stanfield RL, Lyumkis D, Deller MC, et al. Crystal structure of a soluble cleaved HIV-1 envelope trimer. Science. 2013;342(6165):1477-83. Epub 2013/11/02. doi: 10.1126/science.1245625. PubMed PMID: 24179159; PubMed Central PMCID: 3886632.

6. Georgiev IS, Doria-Rose NA, Zhou T, Kwon YD, Staupe RP, Moquin S, et al. Delineating antibody recognition in polyclonal sera from patterns of HIV-1 isolate neutralization. Science. 2013;340(6133):751-6. Epub 2013/05/11. doi: 10.1126/science.1233989. PubMed PMID: 23661761.

7. Freund NT, Wang H, Scharf L, Nogueira L, Horwitz JA, Bar-On Y, et al. Coexistence of potent HIV-1 broadly neutralizing antibodies and antibody-sensitive viruses in a viremic controller. Sci Transl Med. 2017;9(373). Epub 2017/01/20. doi: 10.1126/scitranslmed.aal2144. PubMed PMID: 28100831; PubMed Central PMCID: 5467220.

8. Sok D, van Gils MJ, Pauthner M, Julien JP, Saye-Francisco KL, Hsueh J, et al. Recombinant HIV envelope trimer selects for quaternary-dependent antibodies targeting the trimer apex. Proc Natl Acad Sci U S A. 2014;111(49):17624-9. Epub 2014/11/26. doi: 10.1073/pnas.1415789111. PubMed PMID: 25422458; PubMed Central PMCID: 4267403.

9. Huang J, Kang BH, Pancera M, Lee JH, Tong T, Feng Y, et al. Broad and potent HIV-1 neutralization by a human antibody that binds the gp41-gp120 interface. Nature. 2014;515(7525):138-42. Epub 2014/09/05. doi: 10.1038/nature13601. PubMed PMID: 25186731; PubMed Central PMCID: 4224615.

10. Dreyfus C, Laursen NS, Kwaks T, Zuijdgeest D, Khayat R, Ekiert DC, et al. Highly conserved protective epitopes on influenza B viruses. Science. 2012;337(6100):1343-8. Epub 2012/08/11. doi: 10.1126/science.1222908. PubMed PMID: 22878502; PubMed Central PMCID: 3538841.

11. Oyen D, Torres JL, Cottrell CA, Richter King C, Wilson IA, Ward AB. Cryo-EM structure of P. falciparum circumsporozoite protein with a vaccine-elicited antibody is stabilized by somatically mutated inter-Fab contacts. Sci Adv. 2018;4(10):eaau8529. Epub 2018/10/17. doi: 10.1126/sciadv.aau8529. PubMed PMID: 30324137; PubMed Central PMCID: 6179375.
