# Supplementary material for: A cross-neutralizing antibody between HIV-1 and influenza virus
Source: PLoS Pathog. 2021 Mar 22;17(3):e1009407. doi: 10.1371/journal.ppat.1009407 (PMC8016226; doi:10.1371/journal.ppat.1009407)
Supplement: S2 Table — (DOCX) [file ppat.1009407.s002.docx]

| Residue | | | | | | | | |
| --- | --- | --- | --- | --- | --- | --- | --- | --- |
| Strain | 144 | 145 | 160 | 172 | 192 | 196 | 226 | 246 |
| Mos99 | I | N | R | D | T | T | I | K |
| Pan99 | N* | K | K | E | I | A | V | N* |

**N*-glycosylation site
