# Supplementary material for: A cross-neutralizing antibody between HIV-1 and influenza virus
Source: PLoS Pathog. 2021 Mar 22;17(3):e1009407. doi: 10.1371/journal.ppat.1009407 (PMC8016226; doi:10.1371/journal.ppat.1009407)
Supplement: S3 Table — (DOCX) [file ppat.1009407.s003.docx]

| Strain | Abbreviation |
| --- | --- |
| A/Hong Kong/1/1968 | HK68 |
| A/Bangkok/1/1979 | Bk79 |
| A/Leningrad/360/1986 | Lenin86 |
| A/Beijing/353/1989 | Bei89 |
| A/Shangdong/9/1993 | Shangdong93 |
| A/Panama/2007/1999 | Pan99 |
| A/Moscow/10/1999 | Mos99 |
| A/Wyoming/3/2003 | Wy03 |
| A/Brisbane/10/2007 | Bris07 |
| A/Perth/16/2009 | Perth09 |
| A/Victoria/361/2011 | Vic11 |
| A/Michigan/15/2014 | Mich14 |
| A/North Dakota/26/2016 | NDako16 |
| A/Panama/2007/1999 (N165A) | Pan99 N165A |
| A/Panama/2007/1999 (N246K) | Pan99 N246K |
| A/Panama/2007/1999 (N165A/N246K) | Pan99 N165A/N246K |
| A/Brisbane/10/2007(N165A) | Bris07 N165A |
| A/Taiwan/82486/2014* | A/Tw/82486/14 |
| A/Taiwan/87302/2016* | A/Tw/87302/16 |

*clinical strains from TWCDC
