# Supplementary material for: A cross-neutralizing antibody between HIV-1 and influenza virus
Source: PLoS Pathog. 2021 Mar 22;17(3):e1009407. doi: 10.1371/journal.ppat.1009407 (PMC8016226; doi:10.1371/journal.ppat.1009407)
Supplement: S4 Table — (DOCX) [file ppat.1009407.s004.docx]

| ID | Name | Sequence |  |
| --- | --- | --- | --- |
| 1 | Pan99lib_144_145_F | 5’-CGTACGTCTCAAGGAGATCTAWTAAWAGTTTCTTTAGTAGATTGAATTGGT-3’ |  |
| 2 | Pan99lib_160_F | 5’-GGTTGCACCAATTAAAATACARATATCCAGCACTGAACGTGAC-3’ |  |
| 3 | Pan99lib_172_F | 5’-ACGTGACTATGCCAAACAATGAWAAATTTGACAAATTGTACAT-3’ |  |
| 4 | Pan99lib_192_196_F | 5’-CGAGTACGGACAGTGACCAAAYCAGCCTATATRCTCAAGCATCAGGGAGAGTCACA-3’. |  |
| 5 | Pan99lib_226_F | 5’-CTAGACCCTGGGTAAGGGGTRTCTCCAGCAGAATAAGCATCTA-3’ |  |
| 6 | Pan99lib_160_R | 5’-GTCACGTTCAGTGCTGGATATYTGTATTTTAATTGGTGCAACC-3’ |  |
| 7 | Pan99lib_172_R | 5’-ATGTACAATTTGTCAAATTTWTCATTGTTTGGCATAGTCACGT-3’ |  |
| 8 | Pan99lib_192_196_R | 5’-TGTGACTCTCCCTGATGCTTGAGYATATAGGCTGRTTTGGTCACTGTCCGTACTCG-3’ |  |
| 9 | Pan99lib_226_R | 5’-TAGATGCTTATTCTGCTGGAGAYACCCCTTACCCAGGGTCTAG-3’ |  |
| 10 | Pan99lib_246_R | 5’-CGTACGTCTCAGCAATTAGATTCCCTGTGCTSTTAATCAAAAGTATGTCTCCCG-3’ |  |
