# Supplementary figures and images for: A cross-neutralizing antibody between HIV-1 and influenza virus
Source: PLoS Pathog. 2021 Mar 22;17(3):e1009407. doi: 10.1371/journal.ppat.1009407 (PMC8016226; doi:10.1371/journal.ppat.1009407)

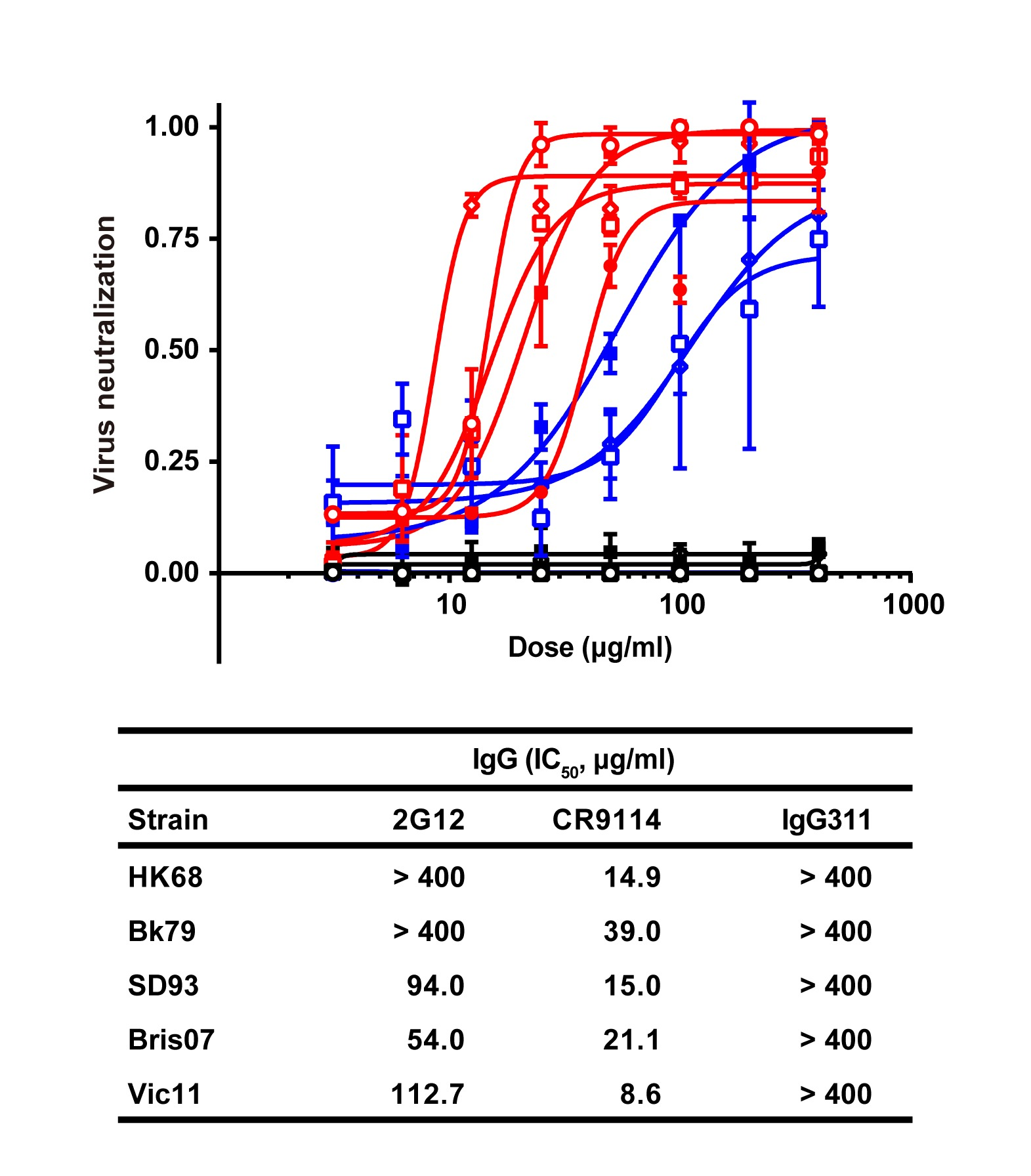

Supplement: S1 Fig — The 2G12 IgG only neutralized contemporary H3N2 influenza strains but not early ones. The anti-influenza virus broadly neutralizing antibody, CR9114 IgG, neutralized early and contemporary virus strains. The anti-malarial antibody, IgG311 showed no neutralizing activity against human H3N2 viruses. HK68, Bk79, SD93, Bris07, Vic11 were denoted by empty circles, filled circles, empty squares, filled squares, and empty diamonds, respectively. CR9114, 2G12, and IgG311 are represented in red, blue, and black. The IC50 > 400 μg/ml indicates no neutralizing activity. (TIF) [file ppat.1009407.s005.tif]

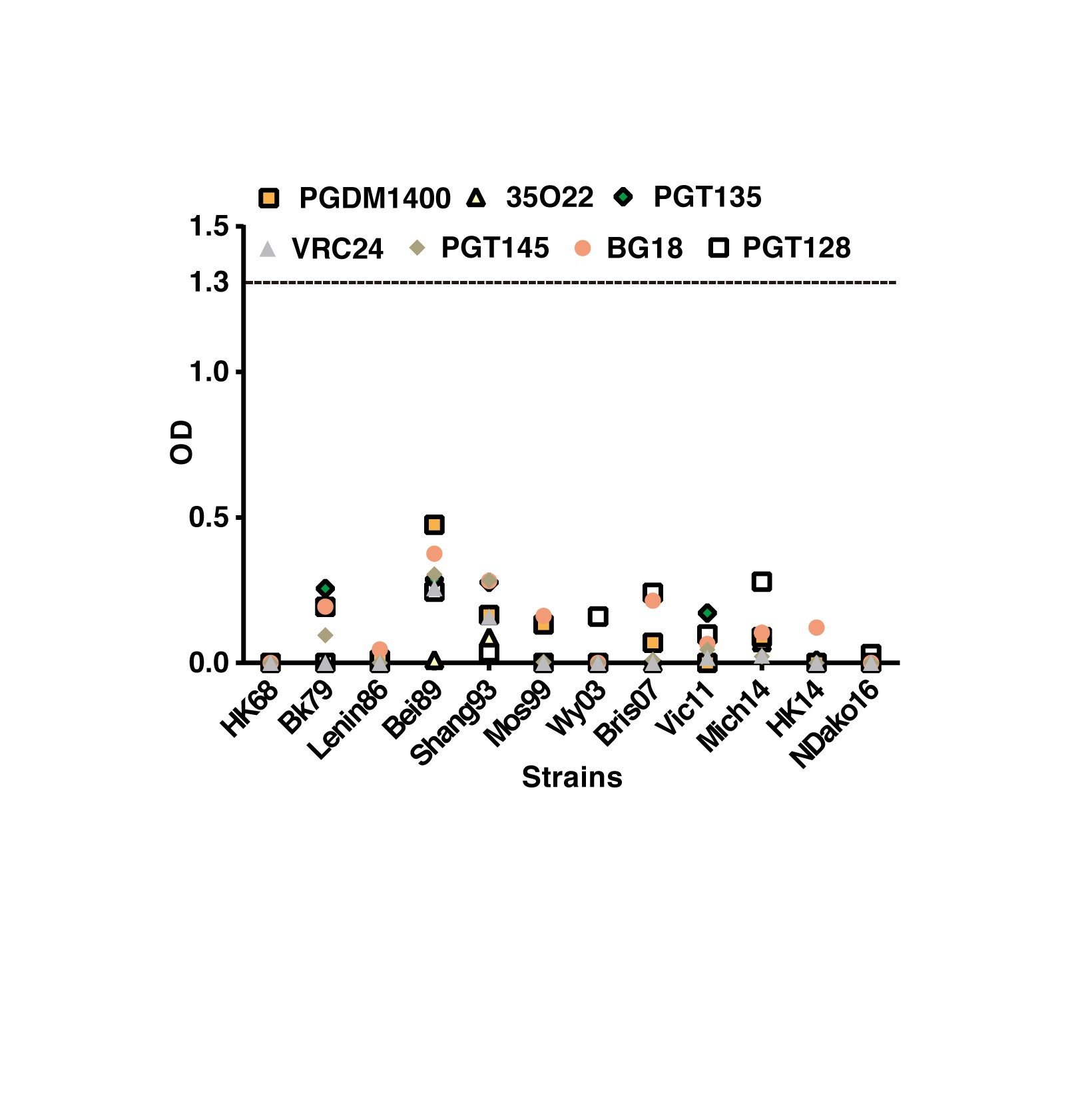

Supplement: S2 Fig — Seven anti-carbohydrate antibodies against HIV-1 do not neutralize any H3N2 viruses at 200 μg/ml in a microneutralization assay. Dashed line represents the OD for healthy cell controls with no virus. (TIF) [file ppat.1009407.s006.tif]

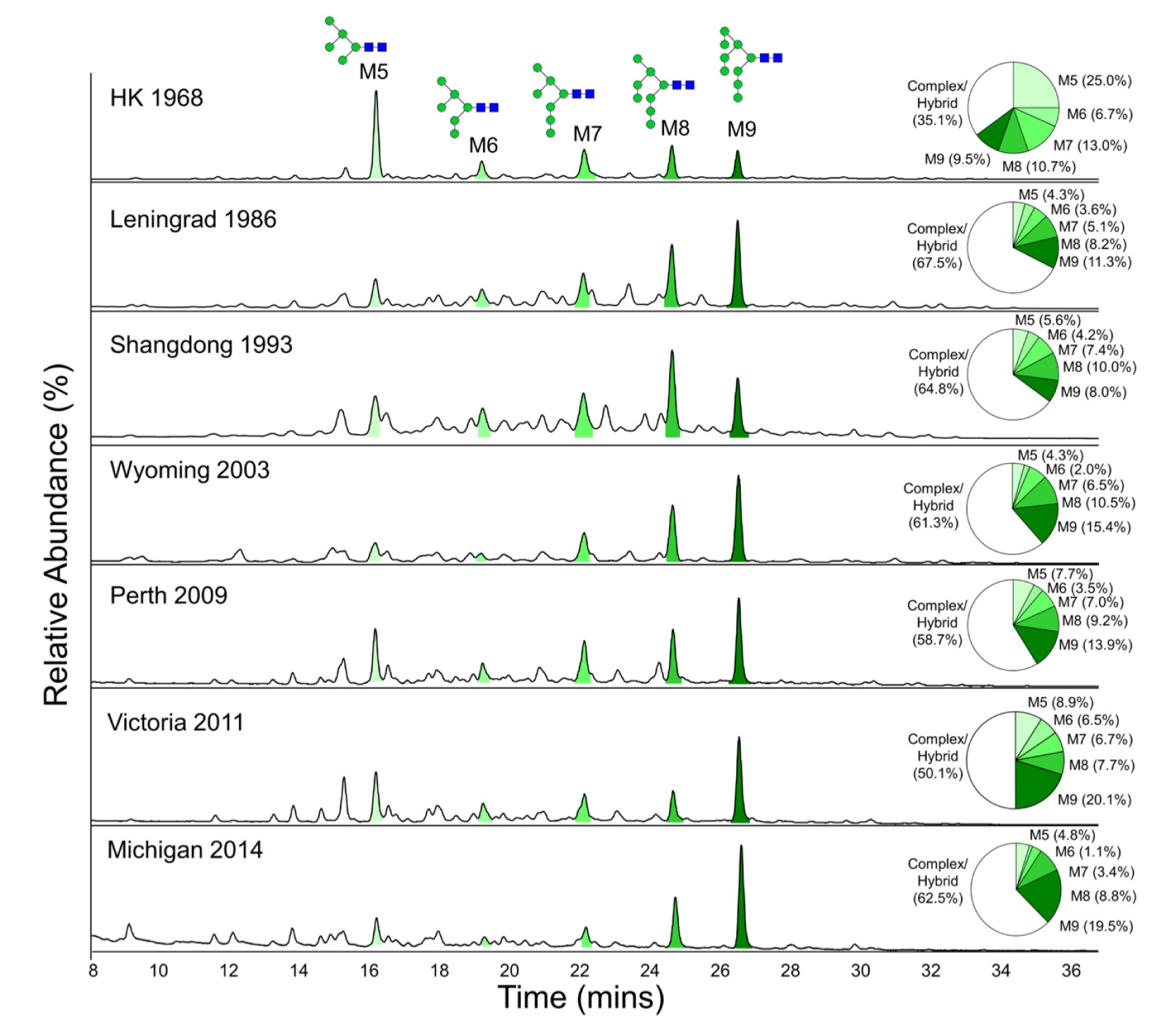

Supplement: S3 Fig — Chromatograms of fluorescently labelled N-linked glycans on HAs from H3N2 strains: HK68, Leningrad86, Shangdong93, Wyoming03, Perth09, Victoria11, and Michigan14. Note the dominant peak of Man5GlcNAc2 on HK68, whereas the 2G12 sensitive strains have prominent Man8/9GlcNAc2 peaks. (TIF) [file ppat.1009407.s007.tif]

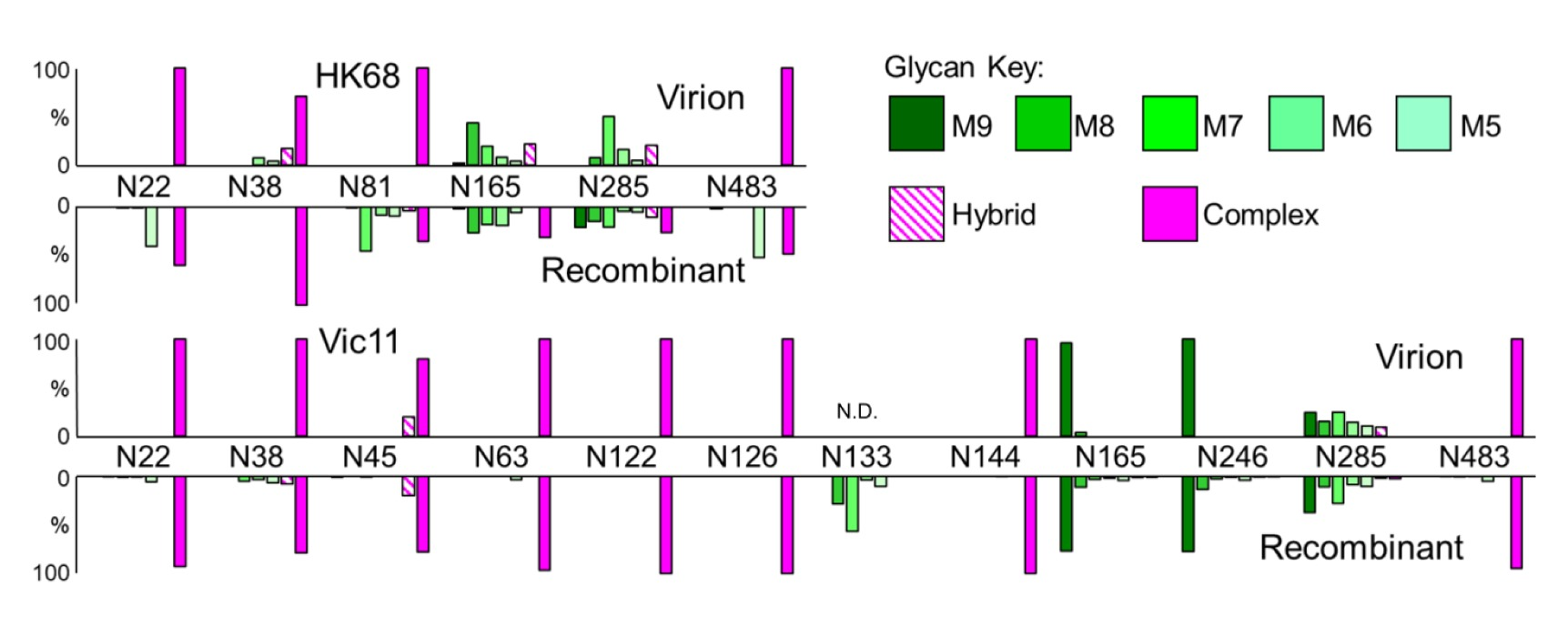

Supplement: S4 Fig — Viral and recombinant HAs from HK68 and Vic11 and their glycans analyzed by LC-MS. Glycans are colored according to the key. (TIF) [file ppat.1009407.s008.tif]

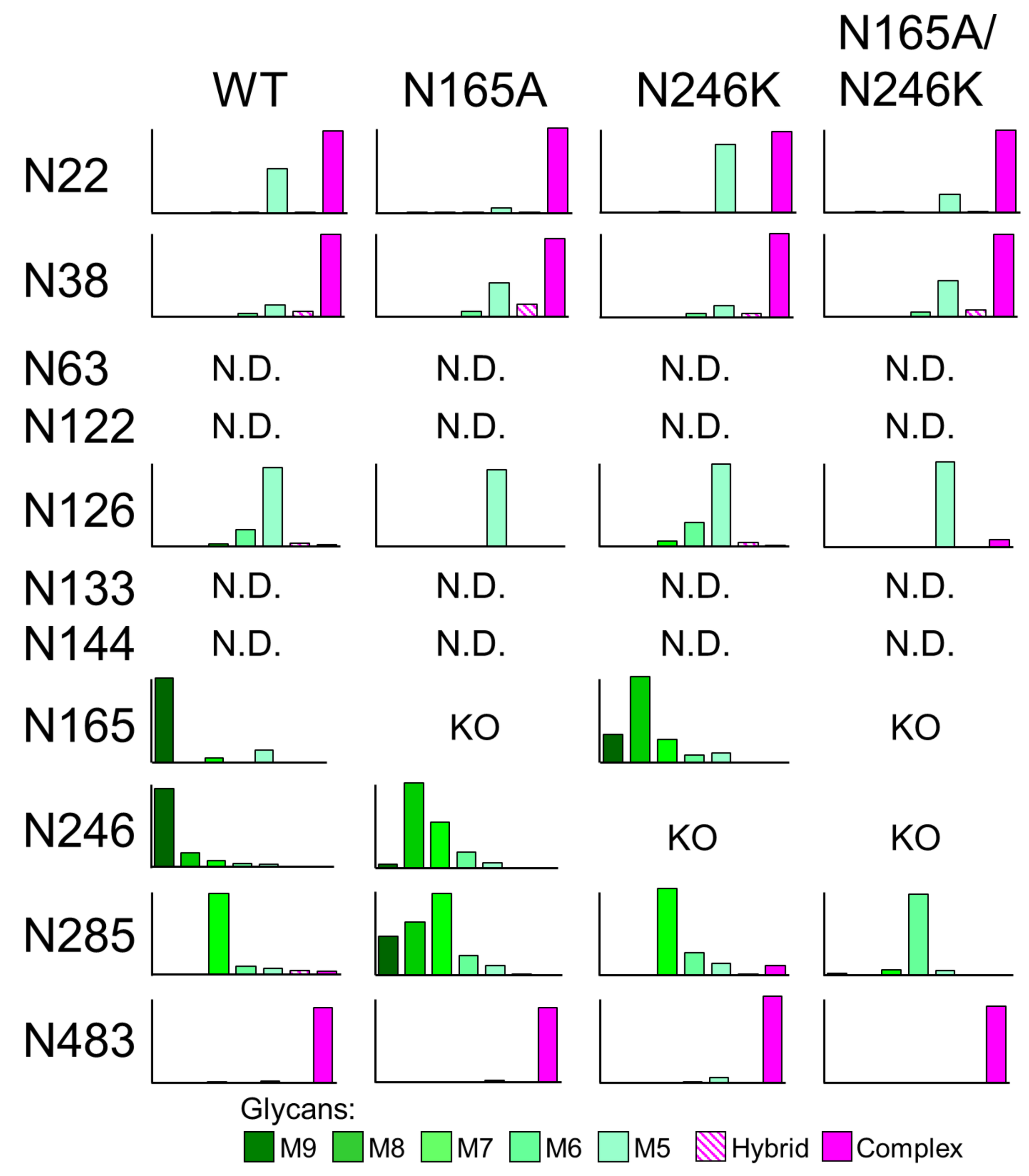

Supplement: S5 Fig — Each N-glycan of the HAs was analyzed by LC-MS. Glycans are colored according to the key. (TIF) [file ppat.1009407.s009.tif]

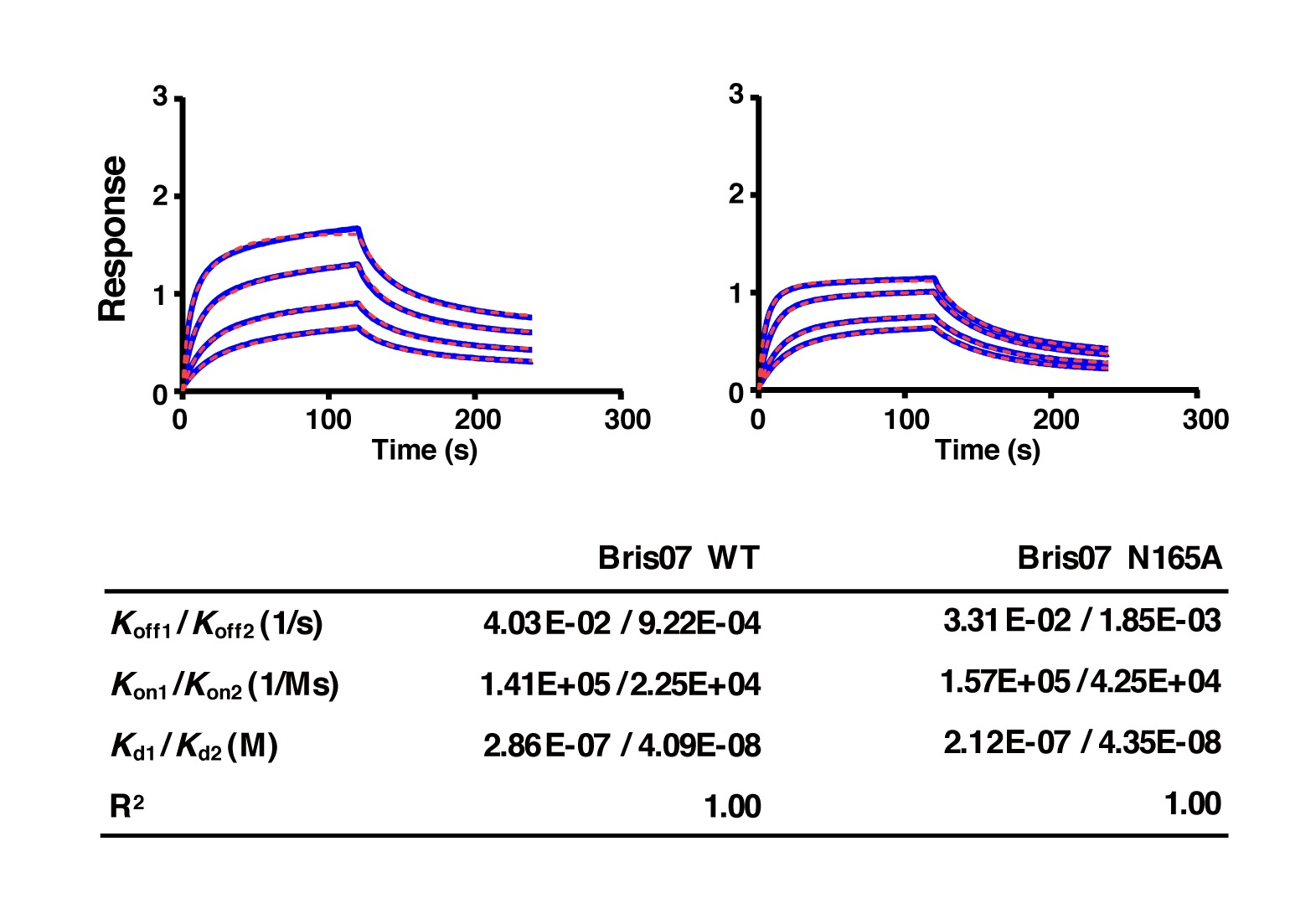

Supplement: S6 Fig — The binding of 2G12 IgG against recombinant Bris07 WT and Bris07 N165A HA proteins was measured by biolayer interferometry (BLI). Blue lines indicate the response curves and red dashed lines represent the fitting curve with 2:1 hetero-ligand binding model for both Bris07 WT and Bris07 N165A. Binding kinetics were measured ranging from 2000 nM to 250 nM by 2-fold serial dilution. The dissociation constant (Koff1/ Koff2), association constant (Kon1/ Kon2), equilibrium dissociation constant (Kd1/ Kd2), and R2 of the fitting are summarized. (TIF) [file ppat.1009407.s010.tif]

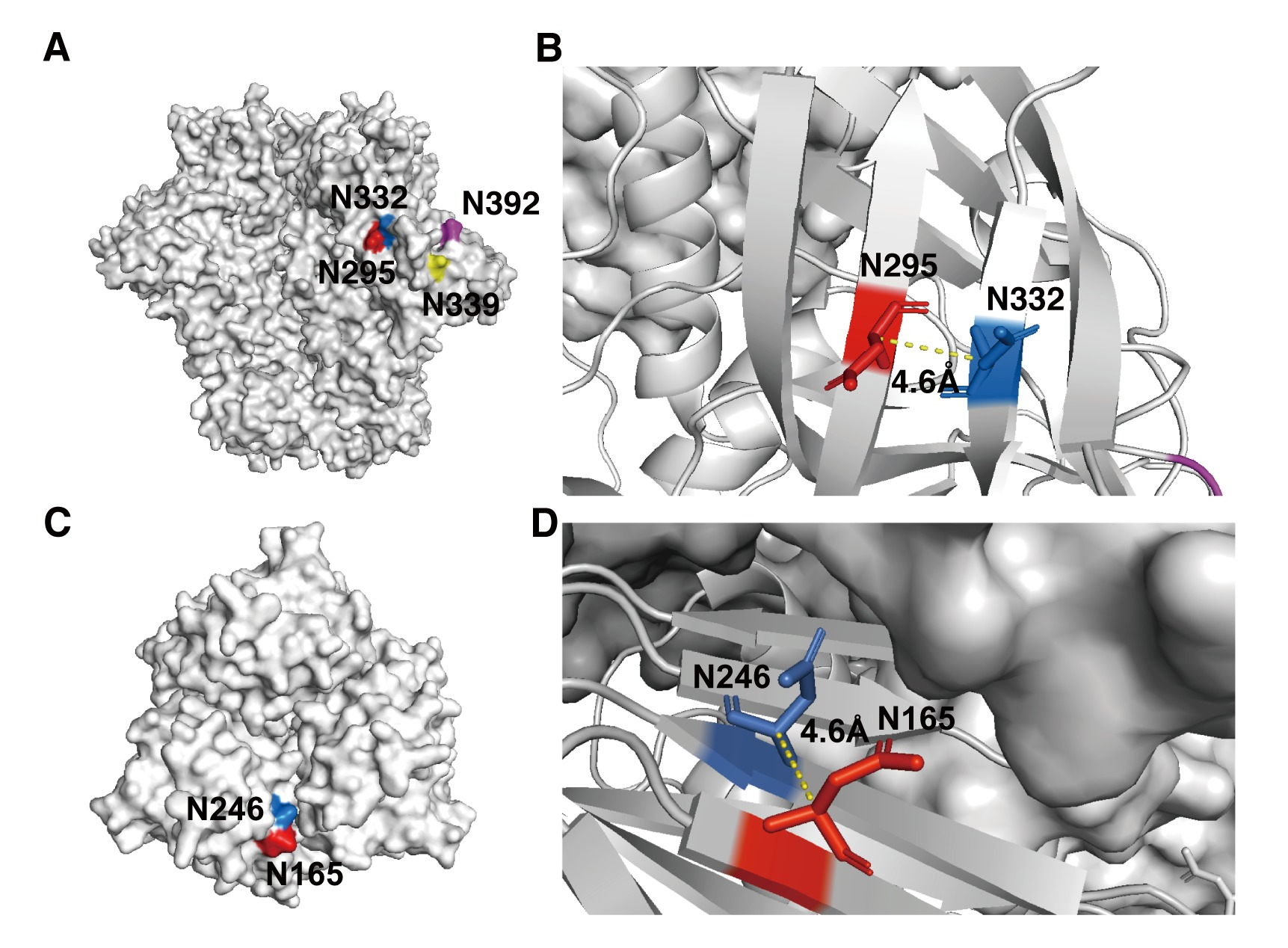

Supplement: S7 Fig — (A) Front view of HIV-1 Env trimer (PDB: 4ZMJ) [97]. Oligomannose on four N-glycosylation sites, N295 (red), N332 (marine), N339 (yellow), and N392 (purple) are involved in 2G12 recognition [11,46]. (B) Close-up view of N295 and N332 on HIV-1 Env. The distance between the Cα’s of N295 and N332 is 4.6Å (yellow dashed line). (C) Top view of influenza H3 hemagglutinin trimer (PDB: 6AOV) [91]. Oligomannose on two N-glycosylation sites, N165 (red) and N246 (marine), are involved in recognition by 2G12. (D) Close-up view of H3 hemagglutinin trimer around N165 and N246. The distance between Cα of N165 and N246 on adjacent strands of a beta-sheet is also 4.6Å (yellow dashed line). (TIF) [file ppat.1009407.s011.tif]

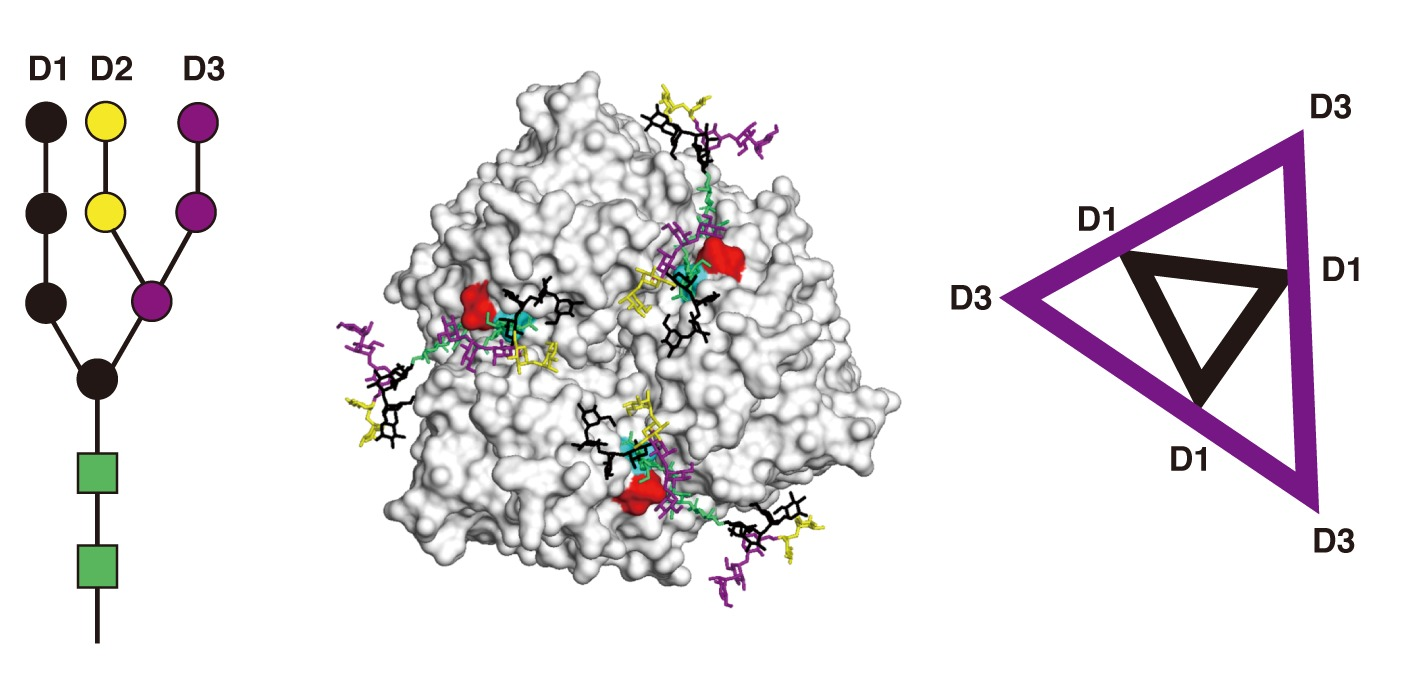

Supplement: S8 Fig — Schematic of Man9GlcNAc2 (left panel). The D1, D2, and D3 arms of Man9GlcNAc2 are in black, yellow, and purple, respectively, and GlcNAc2 in green here and in the other panels. Top view of A/Brisbane/10/07 (H3N2) HA (PDB 6AOV) [91]. Glycans on N165 and N246 were modelled with Man9GlcNAc2 by Charmm-Gui [92] (middle panel). The location of N165 and N246 in each protomer is in red and cyan, respectively. Distances between the D3 arm of N246 to the D1 and D3 arms of N165 are 26 Å and 29 Å, respectively in the same protomer. The right panel illustrates triangles formed by D1 arms (black triangle) and D3 arms (purple triangle) of Man9GlcNAc2 on each protomer of HA. The distances between the ends of D1 to D1, and D3 to D3 of two N246 on different HA protomers are 14 Å and 44 Å, respectively. (TIF) [file ppat.1009407.s012.tif]
